# Barriers and facilitators to healthcare artificial intelligence adoption in Wales: findings from focus groups

**DOI:** 10.3389/fdgth.2026.1927799

**Published:** 2026-10-12

**Authors:** Michal Pruski, Jasmine Rollings, Kathleen L. Withers

**Affiliations:** 1 School of Health Sciences, University of Manchester, Manchester, United Kingdom; 2 CEDAR, Cardiff and Vale University Health Board, Cardiff, United Kingdom; 3 School of Engineering, Cardiff University, Cardiff, United Kingdom

**Keywords:** artificial intelligence, consumer health informatics, ethics, public attitudes, trust

## Abstract

**Background:**

Building on a previous Wales-wide survey, this study aimed to provide more in-depth exploration of the barriers and facilitators to healthcare artificial intelligence adoption in Wales.

**Objectives:**

The study aimed to explore these barriers and facilitators among various social groups, to inform policy development in Wales.

**Methods:**

Four focus groups were conducted with a group of members of the public and a group of healthcare professionals (both groups recruited from a previous survey), as well as a group Sixth Form students and a group of retired adults. Inductive thematic analysis was used to generate themes and to compare the issues raised by the various groups. Contiguity-based analysis was undertaken to underscore how themes related with each other.

**Results:**

A total of 21 individuals participated in the focus groups (healthcare professionals=6, members of the public=6, sixth-form students=5, retirees=4). Seven thematic networks were identified. Across groups, seven themes were identified in the healthcare professional group, six in the general members of the public group, seven in the sixth form student group, and seven in the retiree group. All groups voiced concerns about data privacy and highlighted the importance of human oversight of AI and human contact during the clinical encounter as they expressed more trust in human practitioners. There were though differences between groups, such as greater trust in black-box systems by healthcare professionals, and between individual participants in regard to trust in private organisations developing AI for healthcare organisations.

**Conclusions:**

All groups were concerned with information security and expressed a preference for human contact during the clinical encounter and a human-in-the-loop approach. There were differences between the groups, such as in attitudes towards black-box AI technologies and trust in clinicians. No additional barriers or facilitators were identified for proxy consent.

## Introduction

When implementing digital healthcare technologies, it is not enough that these technologies simply perform well in the tasks which they were designed to undertake. Artificial intelligence (AI) technologies must also possess a broader range of characteristics that make them acceptable to end users. Adoption decisions are made not only on technical grounds, but also on organisational considerations and are influenced by the attitudes of individual actors (1). Those contemplating adopting a technology often consider multiple factors during the decision-making process (2, 3). As such, those developing or implementing AI technologies need to ensure that technologies meet the needs of their target audience, navigating complex ethical problems (4–7).

This study builds on results from a Wales-wide survey and literature review of barriers and facilitators to healthcare adoption (8), adding more depth to this previous investigation. The survey looked the opinions of both members of the public and healthcare practitioners working in a treatment/therapeutic capacity, and split participants into AI sceptics, supporter and the uncertain for the subgroup analysis. Among all groups it identified a preference for an ability to refuse having AI used in one's healthcare, as well as for a greater input of human healthcare professionals into the care process, even if this was at price of improved decision speed that could result from ML applications. Sceptical respondents placed greater emphasis on user autonomy while overall beneficence and nonmaleficence were voted as the most important ethics principles. The most frequently cited barrier among all respondent groups (though particularly so among sceptics) was the risk of unintended/harmful consequences, while the presence of evidence was regarded as the most important facilitator.

Based on literature review carried out in preparation for this study (8), there have been no qualitative studies focused on the context of Wales. While there have been similar studies looking at factors affecting AI adoption among National Health Service (NHS) staff (9, 10), these did not include patients, but focused on healthcare professionals. The only UK study looking at both patients and healthcare staff, that we are aware of, focused only on attitudes towards ethical guidance for healthcare AI use (11).

Looking more internationally, a systematic review by Ahmed and colleagues (12), identified ethical, technological, liability and regulatory, workforce, social, and patient safety issues as the main barriers to healthcare AI implementation, which largely corresponded to the findings from this study. Nair and colleagues (13), had rather different findings, identifying the following barriers: leadership, buy-in, change management, engagement, workflow, finance and human resources, legal issues, training, data, evaluation and monitoring, and maintenance. Surprisingly, as this was a major topic explored in our survey, their literature review did not identify ethical issues as a major finding, which only was identified through subsequent interviews (13). A systematic review of qualitative studies by Martinez-Martinez and colleagues (14) found that the patient-clinician relationship was as key due to the need for empathy, and that it facilitated trust, while AI was seen as being able to increase workflow efficiency. As there were differences in findings from comprehensive studies, it was crucial to assess which factors were most important in Wales.

Finally, little is known about the preferences of the elderly who might be affected by the digital divide, and the under 18-year olds, as well as of the perceptions of the migrant population. In accord with other AI research, we wanted to ensure that voices from diverse backgrounds were heard in the healthcare AI debate (11, 15).

## Study objectives

The purpose of the study was to expand our previous survey findings on barriers and facilitators to healthcare AI adoption in Wales; it was a real-word research project primarily designed to help drive policy change. Our previous literature review, undertaken as background preparation for our survey, identified no Wales-specific studies (8). As such, our first objective was to address this knowledge gap by obtaining qualitative data that would help us with understanding the quantitative results from the survey. As we speculated that different stakeholder groups are likely to perceive different phenomena as barriers and facilitators, our second objective was to ensure that data was collected from various stakeholder groups, to enable a between group comparison. This was also important, as some groups have historically been underrepresented in research and we wanted to ensure representation of participants from across a broad societal spectrum (16). Due to the vast amount of routinely collected patient reported outcomes measures (PROMs) data and the possibility of developing machine learning (ML) algorithms to help guide clinical interventions (17–20), another objective of this study was to collect information to drive future policy work in Wales. Because healthcare is a devolved matter to the Welsh government and the population of Wales varies in many aspects from that of England (21), it would not be appropriate to drive policy in Wales based on data from England.

While at the outset of the study our research, question was: ‘What are the barriers and facilitators to ML adoption in clinical decision-making among different end user groups in Wales?’ following our survey results (8), in which respondents often commented on AI, we decided to permit participants to express their opinions on AI more broadly understood, as long as they were relevant to the other aspects of our original research question.

## Methods

### Stakeholder involvement

The project benefitted from input from two lay representatives and a steering group consisting of NHS Wales staff (clinical, informatics and managerial), civil service and academic members. The lay representatives reviewed a draft of the study protocol, interview guide, the participant information sheet and consent form. The project also benefited from input from a group of lay volunteers from our organisation, as well as a colleague who participated in the survey part of the project but not the focus group part. The study protocol was additionally reviewed by an academic member of the steering group.

### Interview guide

The semi-structured focus group guide (Supplementary File 1) was drafted by MP and reviewed by KW (an experienced qualitative researcher), two lay and two academic members of the steering group, a member of our research team experienced in qualitative research but not involved in this focus group study, as well as by a group of lay volunteers from the authors’ organisation. MP revised the interview guide based on this feedback with oversight from KW. The content of the guide was based on a previously conducted survey, input from a project steering group and identified gaps in the literature. The first four questions focused on understanding the worries and hopes that respondents might have with respect to the introduction of ML technologies in healthcare, and the underlying reasons for these. The fifth question asked participants about how they would balance any properties of such technologies in light of the fact that making a technology more acceptable in one way might be associated with a trade-off relating to another desirable trait. The last question explored whether there were any differences in what made healthcare ML technologies acceptable when the users would be giving proxy consent for someone else as opposed to themselves; this question was partially included to assess whether this was a topic which should be given more attention in future studies. The guide included prompts for the facilitators to refer to the survey results. As this was a semi-structured guide, while the facilitators ensured that all topics were covered, the order differed from sessions to session as the facilitators tried to maintain a fluid flow of conversion guided by the participants. Participants could bring up any topic which was congruent with the overarching research question, as the specific prompts in the interview guide were primarily intended to support the facilitators if the discussion among the participants was stalling.

### Eligibility criteria

To be eligible to participate in the study participants needed to have met at least one of two eligibility criteria. Namely, be either:

- members of the public living in Wales who were at least 16 years old; or
- registered healthcare professionals working in Wales who make treatment or therapy decisions together with or for patients.

### Participant recruitment

Participants expressed their interest by providing their details either after completing a previously reported Wales-wide online survey with the aim of broadening the geographical attendance of participants, or via contact through selected institutions to help with recruitment of groups underrepresented in research. Survey respondents who expressed interest in participating in the focus groups were contacted with additional study information. Participants were subsequently recruited into a general ‘members of the public group’ or a ‘healthcare professional group’ based on maximising attendance at each focus groups. Additionally, to encourage the participation of potentially underrepresented groups in research, three organisations based in Cardiff were contacted: Oasis Cardiff (to recruit from migrant communities), a Sixth Form College (to recruit 16-18 year olds) and a church (to recruit older adults). As such, five focus groups were originally planned. A study advert was placed at the Oasis Cardiff centre, which provides support to migrants and refugees. A staff member of the Sixth Form College approached students interested in healthcare or informatics. An advert was placed in the church's newsletter on two consecutive weeks, which targeted those unfamiliar with digital technologies, and was followed-up by in-person conversations about the study with MP. All participants who expressed interest were given a participant information sheet and filled out a consent form if they wished to take part. MP was the researcher responsible for the recruitment process. Participants received no payment or other incentive for involvement in the focus groups. We did not have access to any background information from medical or other records which would allow us to undertake purposeful sampling.

### Ethics considerations

Cardiff and Vale University Health Board acted as project sponsor (protocol number:8870). The project was registered on the national Integrated Research Application System (IRAS ID:345131). Because of the low risk nature of the study and because it did not recruit patients, IRAS deemed the project to require approval by Health and Care Research Wales which reviewed all study documentation, including the protocol (approval ID: 24/HCRW/0021), but did not require further research ethics committee review. All participants signed informed consent forms before participating in the focus groups (note that in the UK those who are 16 years old or older can provide consent and hence parental consent was not required).

### Focus groups

The focus groups took place between 25 June 2025 and 11 September 2025 and lasted between 90 and 120 min. Each focus group was facilitated by both MP and JR. Face-to-face focus groups (sixth form students and retirees) were recorded on two Dictaphones (Sanyo ICR-EH800D and Olympus WS-853). Focus groups held on Microsoft Teams (healthcare professionals and general members of the public) were recorded using the software's recording function, and the sound-only mp3 file was created using VLC 3.0.3 vetinari (VideoLAN). As face-to-face and online focus groups have different advantage profiles (22), both methods were used to support recruitment and participation. On-line focus groups allowed recruitment of participants from a broader geographical area, while face-to-face focus groups facilitated recruitment of the retirees who might not have been as comfortable using an on-line environment, and of the sixth form students in whose case it was most convenient for them to undertake the focus group at their school.

### Researcher characteristics

MP is a clinical scientist and ethicist, with this being his first major qualitative work. JR comes from a psychology background, with experience in mixed methods research, and compared to the other two authors did not participate in previous parts of this research project. KW is an expert in implementing PROMs in healthcare systems and a very experience mixed methods researcher. KW and MP had an existing professional contact with someone involved with Sixth Form College. MP was known to the church. MP and JR conducted all focus groups, ensuring consistency between focus groups. This also allowed to leverage MP's project knowledge, as well as the fact that JR was more naïve to the broader project and had no pre-existing relationships with any participants helping to ensure objectivity. By not participating in the focus groups KW could better assume a role of an impartial observer when resolving any queries from MP and JR. Reflexivity was primarily ensured through ongoing dialogue between researchers.

### Analysis

After each focus group MP and JR met to compare observations and impressions, and reached consensus on the main observations from each focus group. These observations were used to support analysis of the transcriptions, by highlighting relevant themes which might not come across from the transcript itself. Voice recordings were sent to Essential Secretary (a transcription company), which had a data processing agreement with Cardiff and Vale University Health Board, for ‘verbatim’ transcription. Analysis was undertaken using an inductive thematic analysis approach, rather than a more reflexive approach (23–27), and contiguity-based connections between themes were also explored (28, 29). The focus group methodology was chosen as it allowed the research team to include more participants within the timeframe allocated to the project, the method was conductive to facilitating a potential group consensus and the subsequent generalisation of any conclusions with regards to the four groups to which participants belonged, and because the interaction between participants could help shine light on important issues not directly anticipated by the research team as well as foster a thematically richer discussion (30). The primary aim of the analysis was to provide data to inform policy, and the authors assumed a critical realist stance.

Coding was undertaken using Microsoft Word, and each focus group was coded independently. To ensure consistency and credibility in coding an iterative coding process was employed involving all three researchers to best utilise their backgrounds and various degrees of involvement in the project and with the participants. MP first read each transcript, making initial notes, and then listened to each recording while comparing the transcripts’ accuracy and applying initial codes. JR read each transcript and commented on MP's coding. MP and JR then met to discuss the coding. Next, MP re-read each transcript, finalising sub-theme codes and their grouping within themes. After initial drafting by MP, KW and JR discussed the codes and suggested further grouping of themes into thematic networks to allow for comparability between focus groups. JR suggested the initial thematic networks and their content, which was revised by MP who also standardised theme names between focus groups, performed contiguity analysis. KW helped resolve any queries arising from MP's and JR's discussions.

Previous survey results (8) allowed for the triangulation of the results, as did the results of a public health survey carried out in Wales which asked some questions relating to AI use in mammography image analysis (31). Saturation was assessed post-hoc, as the number of focus groups was pre-determined and each focus group focused on a different segment of population, so assessing saturation would not be an appropriate method of determining if the study should have been terminated at an earlier stage. Additionally, by the end of each focus group participants had no new comments to add, indicating that within-group saturation. Combined with the dialectic nature of focus group and the iterative analysis process involving multiple researchers, this supports the trustworthiness of our findings.

### Data presentation

Throughout the text, quotes are presented with signifiers denoting the focus group from which the quote is taken (HCP—healthcare professionals; MOP—general members of the public; SFS—sixth form students; Ret - retired) and the participant number. The Standards for Reporting Qualitative Research checklist is presented in Supplementary File 2 (32). In the results section, we have presented quotes which we thought best represented the themes across all focus groups. Quotes were initially selected by MP and then discussed by KW and JR for final approval, but we present up to three quotes for each sub-theme in Supplementary File 3 for added transparency.

## Results

### Participant characteristics

Participants were recruited to four of the intended five focus groups: general members of the public, healthcare professionals, sixth form students and retirees. None of the users of the Oasis Cardiff centre volunteered for participation. An overview of participant characteristics is presented in Table 1.

**Table 1:** Participant characteristics.

Group	Number of Participants	Sex	Age	Ethnicity	Notes
Healthcare Professionals	6	2 women 4 men	Youngest in 25–34 category Oldest in 50–64 category	White ethnic backgrounds	5 participants noted AI use in their personal lives on a weekly basis
General Members of the Public	6	3 women 3 men	Youngest in 35–49 category Oldest in 65–74 category	White ethnic backgrounds	Most participants have worked in non-clinical NHS roles. 2 participants worked in the field of digital technologies
Sixth Form Students	5	5 women	All in the 16–18 category (all just finished year 12)	Various ethnic backgrounds	All familiar with AI in day-to-day life and interested in healthcare careers
Retirees	4	4 women	All above the retirement age	White ethnic backgrounds	3 had experience working in healthcare. One used AI regularly as part of her current studies

White ethnic background includes non-British white ethnic backgrounds.

### Themes

A list of themes and sub-themes from each focus group, together with example quotations for each sub-theme can be found in Supplementary File 3. Briefly, seven themes were identified in the healthcare professional group, six in the general members of the public group, seven in the sixth form student group, and seven in the retiree group. Table 2 shows the thematic networks, and what themes from which focus groups fell under these. Note that some themes are present in more than one thematic network because they had a broad coverage within individual focus groups and when considered across all focus groups, they merited splitting to highlight better their individual sub-theme content. As no new themes emerged at the last focus group this indicates that saturation has been reached.

**Table 2:** Thematic networks and their constituent themes.

Thematic network	Thematic network present in	Constitutive themes
Concerns about confidentiality, security and governance	HCP	Confidentiality; Governance; Worries about consequences; Creating trust
	Public	Barriers to trust
	Sixth form	Governance
	Retired	Governance; Data
Worries about the societal consequences of using AI	HCP	Technical understanding; Worries about consequence
	Public	Understanding how things work; Barriers to trust
	Sixth form	Impact on society; AI benefits
	Retired	Treatment recommendations
Trust in organisations and people	HCP	Creating trust
	Public	Creating trust; Barriers to trust
	Sixth form	Organisations; Human element; Governance
	Retired	Organisations
The benefits and advantages of using AI	HCP	AI benefits
	Public	Supporting the healthcare system
	Sixth form	AI benefits
	Retired	Using AI
Consent for the use of AI in care of self and others	HCP	Governance
	Public	Consent to AI
	Sixth form	In care of others
	Retired	Using AI
Errors and bias	HCP	Worries about consequences; Public services not digitally ready
	Public	Barriers to trust
	Sixth form	Errors
Concerns about the NHS developing AI	HCP	Public services not digitally ready
	Public	Concerns about the NHS developing AI
Novelty of AI	Sixth form	AI novelty
	Retired	AI novelty

AI—artificial intelligence; HCP—healthcare professional group; Public—general members of the public group; Retired—retiree group; Sixth form—Sixth form student group. For details on the content of the specific themes please see Supplementary File 3.

Five overarching themes were highlighted consistently in all of the focus groups: governance and data protection, trust, perceived benefits of AI, consent, and the need for human oversight.

### Concerns about confidentiality, security and governance

All the groups had at least one theme that related to this thematic network, which covers the interrelated phenomFena of governance, security and confidentiality. It also highlighted the need for staff guidance on how to use AI technologies and on AI technologies requirements themselves. Data privacy was a highly relevant theme in our previous survey, while healthcare professionals voted the presence of guidance as the top facilitator for healthcare AI adoption (8).

Data concerns related to patient and organisational data; as HCP5 noted ‘*the biggest thing*s *I’ve got at the moment is around confidentiality putting sort of, for example, patient or business sensitive information in AI tools*’. Participants were aware of unethical data use by corporations and that even well-designed systems can fail. Moreover, participants acknowledged the potential negative consequences of such problems ‘*I worry about data leaks, reputational damage and personal liability versus health board maybe’* (HCP5), and proposed measures to minimise such problems e.g., by suggesting that data with patient identifiable information should be kept only on NHS servers, and only anonymised data used with commercial technologies, as well as proposed solutions similar to federated learning. As such, privacy concerns were important for all participant groups, highlighting the importance of employing privacy protective process during AI implementation.

Issues relating to data were broader than privacy, with participants asking if they would have to provide more data than would be collected if AI was not involved and whether ’*somebody's cooking’* the training data (Ret2). This led to the issues of governance of clinical decisions made at the time of the patient encounter, of overall system performance, and the concept of human-in-the-loop. As Ret2 put it ‘*I would like to be sure that there were some safeguards at least to try to make sure that nothing absolutely dreadful went wrong. There's always going to be a hiccup occasionally, nothing in life is perfect, but, you know, you wouldn't want a really big malfunction to cause trouble*’ and ‘presumably there would be some ongoing audit of, of performance?’. Similar sentiments were expressed by the sixth form students, who also highlighted that ‘*humans have more of the chance to take responsibility for it [errors], compared to the machines. So, even if both make, both a human and a machine makes an error, it's more, more likely that a human can take charge and […] make up for it (SFS2)*’. Similarly, sentiments about the need for human oversight, particularly with respect for safety critical tasks were also expressed in the general public and healthcare professional groups.

Staff and organisations need clear guidance on how to use AI to protect them from liability. Healthcare professionals expressed a concern that they received little training and guidance, which makes them feel ‘*quite wary*’ (HCP5) of AI, and that AI implementation cannot happen ‘*without the governance and the legislation of it*’ (HCP3). Conversely, patients need reassurance that they will not end up in a situation where ‘*if you, er, make AI culpable, nobody is culpable*’ (Ret4). Clinicians also expressed a concern about how AI implementation will affect their ability to make independent decisions and their liability because while also expressing concerns regarding their own liability since ‘you can't blame AI, you have to blame an individual’.

Combined with our previous data (8), these last two paragraphs highlight that clear governance process can support staff in confidently using healthcare AI, while additionally reassuring patients about the clinical safety and reliability of the application of such technologies. Importantly governance considerations must include *a priori* technology evaluation and guidance for how the technology is meant to be used clinically.

An important thread, particularly among the sixth form students, related to transparency of AI use as evident from statements like ‘*[patients'] issue doesn't lie with their data being used for improvements but I think it's the, the lack of transparency over the matter*’ (SFS2), highlighting also that there is little opposition to using patient data to continuously improve AI systems.’ Considering the novelty of AI, the sixth form students stated that informed consent to healthcare AI is crucial, at least for the foreseeable time, and that there needs to be transparent reporting on NHS healthcare AI performance. This is consistent with NHS England guidance on the use of ambient voice technologies, which highlight that transparency is particularly important because ‘ambient scribes are a new technology, and it is not reasonable to assume that people will know they are being used without being informed’ (33).

Lastly, there was the issue of whether black-box AI should be deployed in clinical practice, which in our survey was included in the ‘data and algorithmic concerns' grouping which was the fourth most stated barrier. While healthcare professionals were happy to use black box models where one can ‘*basically control what goes in, what goes out*’ (HCP4), members of the public expressed a view that ‘*we just should never go there*’ (MOP5). This highlights an important difference between both groups, but also to highlights the potentially important role of model transparency methods in increasing healthcare AI acceptability (34).

Although all groups expressed concerns regarding safety, privacy and accountability, healthcare professionals tended to focus on practical issues of governance, liability and implementation, whereas members of the public were more concerned with trust and transparency.

### Worries about the societal consequences of using AI

This thematic network covers both the uncertainty of the effect of AI on society and AI's negative effects on society. AI has a certain aura of mystery around it, potentially due to the fact that most people do not understand the technical aspect of how it is created and functions, making it ‘quite hard [for people] to articulate what, what drives that concern’ regarding AI technologies. Fears about AI might also be compounded by the lack of understanding ‘about the level of failure that's currently operating in the world of the NHS’, which might make people perceive AI as a fallible tool being introduced into an infallible system. This theme was not prominent in our previous survey, neither were these concerns concerned in a mammography AI survey carried out in Wales (8, 31).

Broader impacts on society were also mentioned. While the retiree group acknowledged the economic complexities relating to AI making treatment recommendations stating that as ‘*a patient you would choose the most effective treatment […], rather than […] the cost effective, er, but that would be as an individual rather than thinking about the population*’ (Ret4). What was also a concern in this group was that AI might make recommendations that are ethically unacceptable to some people. While healthcare professionals and general members of the public acknowledged the risk of unemployment, they also noted that people can retrain—‘*we just need to change, not deskill, […] if we use the deskilling argument then basically we’re never going to do anything […]It changes the way they work, but it shouldn't be about de-, deskilling*’ (MOP5). Consequently, the advent of AI might require healthcare professionals to adapt to new ways of working, rather than fear job loss.

### Trust in organisations and people

This thematic network focused on which organisations and individuals were trusted by participants, with the need for human contact being a key concept within participants’ accounts. There was also a large degree of mistrust towards private organisations. As Ret2 put it ‘*I would trust the NHS, I’m not quite sure I trust the government and I certainly wouldn't trust these great big corporations like this fellow who's getting skinned alive in America*’ with the group also acknowledging that they would trust ‘*academia*’. When further questioned, a participant noted that the problem is ‘*the possibility of a bias*' that is absent if the organisation is not-for-profit. Sixth form students also corroborated this view, but highlighting that an organisations previous experience was also important. Yet, the opinions were more nuanced among the general members of the public group and the healthcare professionals. They highlighted that the public sector is not very good at innovation and that technology development would be much faster if it was managed by private organisations. Yet, they still acknowledged problems with private organisations ‘*because it's political […] or worse still, giving you answers that are clearly prejudiced, based on that company's current thinking*’ and that they would ‘*be slightly less worried if those black boxes were being developed, erm, and, and owned within the UK than I would if they were owned in the US*' (general member of the public) (MOP6). This corresponds to our survey findings highlighting that the NHS and academia were associated with greater trust than private companies and the government/civil service, while there was recognition among survey respondents that the NHS can be riddled with bureaucracy and that not-for-profit companies could offer the optimum of trust and ability to perform well in developing healthcare AI (8).

While sixth form students expressed almost unreserved trust in healthcare professionals the other groups had a more balanced view of healthcare professionals. MOP1 noted how radically different the care they received from healthcare staff was in two different organisations and how this affected their trust in these organisations ‘*I’m very lucky I have got a specialist hospital that looks after me and they’re very, very, erm, clear in what they do and one, one thing and another. However, my local health board is very different and I don't have trust with those for various reasons’*. Such trust is important for health services rolling out AI technologies because, as a member of the public put it, ‘*I would be quite happy to be a guinea pig for that because I, I have full trust [in surgeons from the specialist hospital]’*(MOP1), highlighting that people not only need to trust the technology, but also in those who implement and use it. Indeed, within the framework we used to develop our initial survey (8), we treated trust in clinical AI (by patient and clinicians) as the outcome as the outcome of a well-managed clinical technology implementation.

Perhaps most strikingly with respect to this theme is the importance of face-to-face contact during the care process—as Ret2 noted, ‘*It's got to be face to face, there's no substitute for a real human being*.’ Participants in general did not want to interact with chatbots or have phone consultation, though they noted some exceptions, and thought that clinicians can see beyond the raw data, assess the patient holistically, and see the clinical exceptions. Members of the public wanted the introduction of AI to be an opportunity to have more face-to-face time with clinicians—‘*it would be nice if we could actually change that, where the GPs have more time with the patient instead of, you know, like I said, that ten minute thing and, you know’* (MOP1). Healthcare professionals also wanted to preserve this human element in the clinical encounter and ensure that they ‘*would make that final decision with the patient’* (HCP6). This was in agreement with our survey results, where the preference for humans remaining in charge and the need for person-centred care were amongst the most prominent themes highlighted by respondents (8).

#### The benefits and advantages of using AI

Despite most participants being cautious about the introduction of healthcare AI, most could see positives to it, too. This is particularly important as beneficence was previously voted as the most important ethics principle in healthcare AI implementation (8). Three of these positives stood out.

The first is the ability of AI to be more accurate than humans. As HCP3 noted ‘*my point is not to be naïve to, to know that there is, there is scope for artificial intelligence to be more accurate than humans in some degree*’. Within this context, SFS4 specifically commented on image analysis where ‘*I think it's very easy to, erm, for a health care professional to see a scan and make, er, a diagnosis but miss, you know, a certain section of it that kind of changes everything, […] I think AI would help enhance focus on certain things. Like a diagnosis that's harder to make*’. This sentiment was also expressed in mammography AI survey where most respondents believed that the use of AI would be associated with improved accuracy of results (31).

The second, is the ability to save time on simple tasks or extend service provision. As HCP4 noted ‘*AI is working like your assistant to automate things, to speed things, to save the costs*' and HCP5 noted that ‘*I think the classic for dieticians is every patient comes to see us and they want a diet plan, they want a meal plan and we don't really, you know, don't al-, don't always do that, erm, but actually AI can do that in seconds and give something really tailored to the foods they like based on their conditions’*. Similarly, a member of the public highlighted that AI could lessen admin burden without introducing significant risks. Indeed, speed of the return of results and efficiency were the two most highlighted benefits of AI in the mammography survey (31).

The last is the ability to counter some of the human biases. SFS3 highlighted that AI ‘*would be more objective but it, because obviously it has no bias on gender’*. Similarly, HCP5 noted that ‘*as clinicians we’ve got our own biases […] an AI facilitated decision or treatment plan, for you to table that alongside everything else and then, as part of a best interest decision, I think it could just give a different perspective, different angle*.’ Nevertheless, a further section will provide a different perspective on the issue of bias.

### Consent for the use of AI in care of self and others

This is the final thematic network that was present in all four focus groups. There was broad agreement that the general standards of consent should apply to healthcare AI, including when decisions are made for patients lacking capacity. As HCP1 highlighted ‘*all the rules apply whether you use AI or not and so therefore your decision making shouldn't be any different.’* The expressed views highlight participants’ corroboration with standard best-interest decision-making, such as that decision-makers need to be aware of the patient's wishes. Interestingly, SFS3 highlighted that they would balance the wishes of the person with the medical benefit of an intervention—‘*It depends on how much it could help, because if it was just speeding it up or something I, I’d still say no, but if it was like the AI can definitely do it, and there's absolutely no chance that people can, then I would say yes. I wouldn't be happy about it but, I, I, in that situation you’re kind of forced to’*, highlighting the complexity of best-interest decisions. Issues of proxy consent was a novel theme that to our best of knowledge has not been previously explored.

Regarding consent, the healthcare professional group noted that consent would not be needed ‘*if it's facilitating a decision and you’re, as a clinician, taking that decision, that's fine and we probably, you, you know, you probably wouldn't necessarily cite all the sources we’ve used’* (HCP5). Furthermore, it was highlighted by MOP3 that refusals to some uses of AI might not be practical ‘*I would love to say we should absolutely be able to say you cannot use AI in my treatment […], but then I think, well, you wouldn't turn around and say, oh no, I, I don't want to see this particular professional if that is the professional who needs to deal with you[…], you wouldn't say, oh I don't want my thing to be sent* via *Outlook or I don't want this record being shared with other members of the clinical team*.’ This highlights the complexity of the discussion surrounding consent to clinical AI, where while there is agreement that patients should have a right to refuse AI use, implementation of this might not be practically straight forward and explicit consent for AI sue might not be necessary (8, 35–37).

### Errors and bias

With the exception of the retiree group, all other groups had at least one sub-theme relating to AI bias, be it data bias or automation bias, or error. It was somewhat surprising that this thematic network did not span all four groups as nonmaleficence was the second most important ethics principle in our survey, with unintended/negative consequences being the top barrier (8). This might be because this thematic network has a large overlap with the ‘concerns about confidentiality, security and governance’ which also consistent of subthemes relating to nonmaleficence and did include content from the retiree group.

Participants were aware that issues of bias are historically embedded in healthcare, rather than being a novel phenomenon introduced by AI, and that bias is ’*something that if we don't fix that, that is something that would just end up being, it would end up being, it ended up going on whenever you use Machine Learning’* (MOP2). As to automation bias, SFS1 stated that the problem stemmed from ‘*humans we like to make things easier for ourselves*’, which as MOP6 noted might result in an attitude by clinicians that since AI gives ‘*90% of the right answers why should I worry about the other 10%*’. This might result in deskilling—‘*becoming too dependent on it [AI] and losing my ability to critically think and critically judge situations and, and that's priceless*’ (HCP6).

With regards to AI errors in healthcare, SFS3 noted that ‘*I think it's worse if the machine makes an error just because you, you do expect AI to be all knowing sort of and […] being wrong in medicine is like a big deal, it could be risking lives’*. With regards to preventing AI errors, members of the public also expressed worry about how AI will evolve, especially if different AI systems will interact with each other, which might create errors that will be difficult to weed out during early phases of technology implementation, particularly in relation to black-box AI—‘*That's the massive difference, that if paracetamol was an AI, you’re most likely to find in five years’ time, not only is it doing your pain management, oh, but it's also doing this. We’re not quite sure why but hey, it's a good thing, so we’ll just leave it be […] then the outcome from that algorithm maybe fed into other ones and […] this is the problem, where you build up a network of them, […] weights that is used by those, those different outputs that we don't fully understand why it applies them in certain aspects and not others.*’ (MOP6)—and has consequences for the safety testing of such systems—‘*the amount of test, retesting you should do would probably be too much. It would then hinder the AI in being able to move forward*’ (MOP6). Moreover, NHS systems might not be ready for safely and effectively implementing AI technologies because AI ‘*needs accurate detailed information and […] we’ve got paper records […] you need an electronic system […] we need the basics first before we get AI to help us with any clinical decision making*’ (HCP1).

This section highlights that participants appreciate the complexity of the problems relating to healthcare AI implementation. Particularly important is the realisation that good AI depends on good data, and that collecting good data is a broader problem in itself and affects healthcare independently of AI.

### Concerns about the NHS developing AI

The concerns of healthcare professionals and the general members of the public regarding the NHS developing healthcare AI are best summed up as ‘*We need innovation to actually happen. It's not going to happen inside the NHS*’ (MOP5). As HCP2 noted ‘*I think private companies can pay more so they’ll attract people who are the best of the best, […]whereas I think government departments, erm, typically don't pay as much as private companies,’*. This is also linked to the low digital maturity of the NHS ‘*the public probably don't understand how poor the NHS is currently in terms of its use of technology and data. Forget about AI, the level of integration, the level of knowledge the level of failure through to manual processes that doctors and nurses have to deal with and then the public have to deal with is, is, is not understood*’ (MOP5). Moreover, a member of the public highlighted problems with resources and questioned whether such AI development is the purpose of the NHS. The fact that these two responded groups contributed to this network might reflect their awareness of the current resource struggles faced by the NHS, of which the retirees and sixth form students might not be so aware.

### Novelty of AI

Sixth form students and retirees placed a lot of emphasis on the novelty of AI. As highlighted earlier, the novelty of AI was expressed as one of the drivers for the need for transparency. They noted that at the core of this novelty was the independence from human oversight ‘*maybe people are considering the possibility of it, one day there won't be a person looking after the AI and the AI will do everything on its own. Maybe that's why we’re talking more about it’* (SFS2). This novelty meant that ‘*at first, like, [healthcare AI] should be voluntary’* (SFS3). Yet, time will create familiarity—*‘I feel like so many like technologies that like 20 years ago, people were like this is so new, how are we meant to trust it, but now nobody talks about it because it's standard now’* (SFS4). They also highlighted that particularly older people might be hesitant about AI.

While some retirees admitted worrying about AI novelty, one participant stated that these might be simply ‘dinosaur thoughts’ (Ret1). As the discussion progressed, a Ret1 noted that ‘I came in feeling very sceptical about all of this and now I feel, oh, there's a lot more to it and it makes a lot more sense because it's just got more information’. Perhaps an important part in shaping opinions about AI was their ability to draw analogies with technologies familiar to them, with Ret3 noting that AI ‘is kind of the computer equivalent of a Cochrane review, isn't it?’

This is an interesting intergenerational juxtaposition and highlights that while concerns about the digital divide are important, the ability of the retired to deal with technology should not be underappreciated. It is also highlights that policy devised now might have to change if AI becomes more ubiquitous, as highlighted in the ‘concerns about confidentiality, security and governance’ section discussing consent.

### Interviewer observations

During the focus groups, we made some observations that deserve particular emphasis.

The first observation relates to participants’ understanding of AI. While some participants explicitly stated their lack of technical understanding of AI, in other cases this was apparent from the way in which participants talked about AI. Participants did not always distinguish between such modalities of AI as ML and large language models, or appreciate how ML models are trained compared to large language models. Large language models seemed to have been primarily on the forefront of participants’ thoughts, despite us using a machine learning example at the start of each focus group (Supplementary File 1). Interestingly, a member of the public highlighted this common lack of differentiation between AI modalities. This was congruent with previous survey findings, where despite attempts to focus participant on ML, large language model considerations also appeared to dominate the responses (8).This preference for discussing large language models should be regarded as a finding in itself, as it is suggestive of what is the key concern for the participants. Somewhat surprisingly, the retiree group exhibited the greatest awareness of needing to focus on ML applications (as per the interview guide; Supplementary File 1), though still commented on other healthcare AI applications.

The second is the extent of trust in healthcare professionals placed by the sixth form students. Though this was included in the ‘trust in organisations and people’ thematic network, we believe that the extent of this can only be appreciated through the totality of their responses. This might be a reflection of a relative naivety of this group with respect to healthcare exposure. Yet, this should not be dismissed as an important finding, as it also likely shows how this group interacts with the healthcare system.

### Interrelationships between thematic networks

There was clear overlap between several of the thematic networks, which became apparent during the coding process when MP and JR realised that some sub-theme could potentially fit under more than one theme. As such, interrelated themes appeared in multiple thematic networks, crossing over at focus group and sub-theme level. Particularly prominent in this regard was the ‘concerns about confidentiality, security and governance’ thematic network. It overlapped with the ‘trust in organisations and people’ thematic network in regards to issues of staff training and the responsibility that organisations bear in relation to mistakes their AI might make. This also relates to the thematic network of ‘errors and bias’, as governance arrangements relate to the issues of legal liability when errors occur. Furthermore, issues of transparency related to both ‘AI novelty’ and ‘consent for the use of AI in care of self and others’. In the first case, this is because a lot of the drive for transparency was due to the novelty of these interventions and because it was on the minds of the public. In the second case, it is because the validity of consent is linked explicitly to the consenting party being informed, which cannot happen without transparency. Interestingly, in a previous survey carried out in Wales, transparency was not such a highly rated principle among respondents (8), but transparency among focus group participants appears to be a mechanism for operationalising the principle of autonomy, which was highly rated in among the survey respondents. Finally, ‘trust in organisations and people’ overlapped with ‘concerns about the NHS developing AI’. This interaction resulted in an interesting contradiction, as while participants expressed a higher level of trust in NHS organisations, they also highlighted that private institutions might be better placed at developing clinical AI, despite the worries about data privacy expressed in the ‘concerns about confidentiality, security and governance’ thematic network. As mentioned earlier, this largely matched previous survey findings, where respondents from Wales expressed greater trust in NHS and academic institutions to develop AI, compared to private companies and the government, while also acknowledging the advantages that non-public institutions have (8). Some of the interrelations between thematic networks are presented in Figure 1.

**Figure 1:**
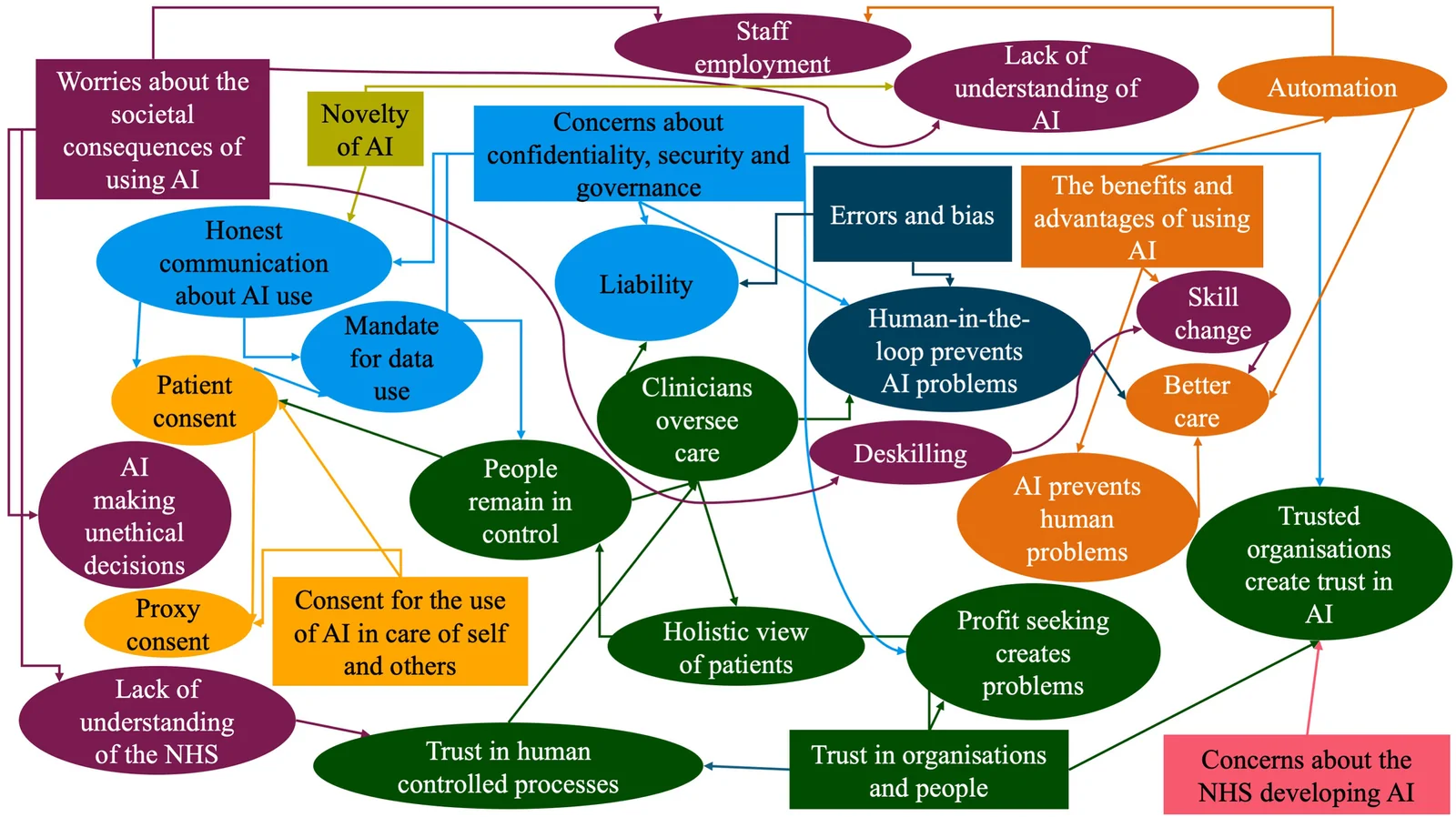
A simplified representation of some of the interrelations between thematic networks. Thematic networks are presented in rectangular boxes, with each presented in a different colour scheme. Ovals represent some of the findings related to the thematic networks (represented by the ovals’ colour schemes), but for simplification purposes oval names might not correspond to theme names. Ovals use only a single colour corresponding to the main thematic network they relate to rather than a mix of colours of all the thematic networks to which they relate. Arrows represent important influence of the parent item onto the target item; arrow colours reflect the parent item from which they originate

The recurrence of the five major thematic networks across all four participant groups and the absence of new themes in the final focus group suggest substantial convergence in stakeholder perspectives regarding healthcare AI. Taken together, the findings suggest that acceptability of healthcare AI is shaped less by the technology itself than by the context in which it is deployed. Across participant groups, trust was closely linked to transparency, governance, accountability and the continued involvement of healthcare professionals in decision-making. Participants generally viewed AI positively when it was perceived as enhancing accuracy and efficiency, but concerns arose when AI was seen as reducing human oversight or obscuring responsibility for clinical decisions. These findings indicate that successful implementation of healthcare AI will depend not only on technical performance but also on transparent governance, public engagement and preserving the human elements of healthcare delivery.

## Discussion

### Study focus limitations

While the study originally intended to focus on ML within the context of prudent healthcare, participants often discussed topics relating to AI and healthcare in general, potentially due to a lack of conceptual separation between the different types of AI. This also occurred during the survey stage of the project, and is potentially associated with the rise in prominence of large language models (8). MP and JR did not stop participants from broader discussion, though they tried to steer it back onto the topic of ML and prudent healthcare when opportunities arose.

While the focus deviation could be seen as affecting the study's credibility, it was congruent with our survey results, allowing for triangulation of results. Moreover, the interview guide made explicit reference to discussing the survey results, and even if participants did not bring broader healthcare AI into the discussion, we would still have asked them to comment on it on this basis. Additionally, this study was treated as an opportunity for respondents to participate in the co-production of healthcare services, which is one of the principles of prudent healthcare adopted in Wales (38), and we did not want to deprive respondents from an opportunity to voice important concerns about how digital services should be delivered in Wales. Finally, it was anticipated that conversation about AI in healthcare would still reveal pertinent points to ML in the context of prudent healthcare and broaden the applicability of the results to broader healthcare AI implementation in Wales, e.g., the result could support future debates about the use of ambient voice technologies (39). Yet, this meant that less time was dedicated to ML specific considerations (e.g., those about balancing specificity and sensitivity). As such, this deviation from the original study question should not be seen as risk to the study's integrity, but as a commitment unearthing what is important to those living and working in Wales.

### Participant recruitment limitations

The focus groups were undertaken during normal working hours. This limited the opportunity for attendance of those with other work commitments.

While the project aimed to recruit participants who would usually not partake in such studies, this was achieved with only limited success. We were unable to recruit anyone from the migrant/refugee community despite support from the Oasis Cardiff centre. This was potentially due to lack of financial remuneration for participation and lack of advertising of the study in another language than English and Welsh, though the study team attempted to advertise the study in Arabic, but were discouraged from implementing this due to the research approval process. While it was hoped that it would serve primarily to recruit those with low levels of digital literacy, this proved rather challenging and the final sample would be better described as retirees, although several participants described themselves as having little knowledge about digital technologies.

Even among the general members of the public and retired groups, a high proportion of participants were from a healthcare background, whether as retired clinicians or working in the NHS in non-clinical roles. As such, there are large limitations as to the way these focus groups truly represented the views of the lay public.

As we did not have access to any personal records, we could not undertake purposeful sampling. As such, using the combined approach of utilising past survey participants to aid geographical representation and contacting organisations from which we were more likely to recruit participants from research-underrepresented social groups was a pragmatic approach taken to minimise recruitment bias.

### Between group comparison

The presence of a group of sixth form students and a group of retirees provided an opportunity to contrast intergenerational attitudes. Similarly, it seemed opportune to contrast the healthcare professional group with the general members of the public group, especially given the latter's experience with digital technologies, and non-clinical experience of the NHS. This helped to build on the attitude-based subgroup analysis in our survey (8), though none of the groups could overtly identified with a pre-specified attitude towards AI. Despite self-described openness towards healthcare AI, our impression was that the sixth form students were the most AI-sceptical group.

#### Healthcare professionals and members of the public

The first striking difference between both groups was that the members of the public have shown great animosity towards black-box AI, while the healthcare professional group did not mind black-box AI, but wanted to have control over the inputs and the ability to assess the AI's outputs. Nevertheless, the issue of how to validate the output of a pipeline of several black-box (or for that matter any AI) technologies is an important matter for clinical safety consideration and device regulation (40). The second notable difference was that in the healthcare professional group, issues relating to how much input humans should have in the care process were more prominent than in the members of the public group, although the members of the public group did discuss such concepts as human-in-the-loop, and the importance of face-to-face contact with a clinician. There was an agreement between both groups about allowing the use of anonymised data to improve systems.

#### Sixth form students and the retired

Sixth formers appeared very confident in clinicians’ ability, and were wary of AI despite declared openness about it. The retired group, while overall having a high level of trust in NHS healthcare staff, were more open about acknowledging the potential of human errors in healthcare. The retirees, while appearing to have less experience with AI, appeared more optimistic about the potential of AI to aid clinicians. The students also thought that there was an important epistemic difference in the process governing diagnosis versus those of treatment. Yet, both groups were concerned with data privacy and the ulterior motives of private companies developing AI technologies.

### General discussion

#### Key areas of consensus

There was broad consensus among the four groups that preserving human contact during the clinical encounter was important, which was also a key finding from a previous survey conducted in Wales (8). Yet, the motivation for this differed between groups. Among the sixth form students, the desire to have input from human clinicians seemed to relate to the trust that participants had in clinician's skill and knowledge, and the belief that AI would not be able to reflect upon its potential mistakes. They also noted that clinicians would be able to tease out relevant information from a patient, while an autonomous system would rely on the patient supplying the correct information themselves. Among healthcare professionals, while there was some recognition that the use of AI could enhance practice, there was a belief that clinicians still brought a certain amount of skill into the decision-making process, as well as the human element of an interpersonal interaction. In this group, there was also an element of maintaining control over the therapeutic encounter which could potentially protect the healthcare professional from responsibility for actions that would otherwise be autonomously taken by the AI and with which the healthcare professional would not agree, which might correspond with the desire for clear guidance and liability criteria expressed by healthcare professional in our survey (8). Members of the retired group also recognised that there is a specific human element to the patient-clinician encounter that could not be substituted by the technology. Moreover, they noted the importance of a first impression (end-of-bed) assessment, where the clinician gains a lot of information about the state of a patient just by seeing their overall demeanour, which would not be possible for an AI. Members of the general public group felt that human presence in the care process would act as a safeguard, and that the point of using AI was to allow clinicians to focus more on patients during clinical encounters. Some of these points are currently the subject of the debate surrounding AI scribing tools. For example, while some highlight that AI scribing technologies reduce clinician burnout and allow clinicians to focus on patients, there are also concerns that while AI scribes might improve notes for continuity of care purposes, especially when patients receive care from multiple clinicians, they might negatively affect clinicians’ recall of previous encounters with the patient and hence negatively affect continuity of care when the same clinician sees the patient (41–44). Outside of the context of scribing tools, there is a concern that ML-related needs for accurate data entry might contribute to clinicians’ focusing more on data entry during the clinical encounter than on interacting with patients (45).

Data privacy was another key finding, mentioned in all groups, although the most comprehensive discussion regarding this was in the healthcare professional group. Interestingly while we previously found data privacy concerns to be of particular importance to respondents (8), another survey carried out in Wales found that most respondents did not believe that the introduction of AI in mammography imaging review process would have any impact on privacy (31). Here, findings relating to data privacy included concerns regarding both individual patient data and organisational data. Through suggesting ideas similar to federated learning, and distinguishing data use within NHS systems and when sent to external servers, it is clear that practitioners are actively looking for ethical solutions to deliver healthcare benefits while minimising risks. Data privacy issues are not limited to AI technologies, or even digital care itself. The extent of AI-related data risks is unclear considering the core issues of poor NHS digital infrastructure and the digital hygiene of some healthcare staff, which is not a Wales only problem (46). It is though reassuring that healthcare professionals express similar concerns to members of the public.

One of the issues explored in this work, which so far received little attention, was how participants viewed proxy consent in the context of AI-augmented care. Participants were asked whether they would be making decisions differently if they were acting as a proxy for family members or friends, or if they thought that any special governance considerations would have to be in place for patients without capacity (in the healthcare professional group). There was broad consensus between groups that participants would utilise the same thresholds for acceptance of an AI as they would use for themselves, but they would also take into account the general AI attitude of the patient, and refuse proxy consent if they knew the patient was AI averse. In the sixth form student group, there was a bit more nuance to this, with one participant stating that if only the AI could undertake a life-saving intervention, then they would provide proxy consent even if they knew that the participant was AI averse, but would not give consent to AI use if it would only improve care process. Perhaps this later case is congruent with the fact that patients might change their attitude towards an intervention in light of new knowledge about the risks of agreeing and not agreeing to an intervention. As such, participant responses suggest that AI-augmented healthcare interventions do not pose new challenges with respect to proxy decision-making. Finally, participants highlighted that there might be potential practical challenges in refusing consent to the use of AI in healthcare if these technologies become ubiquitously embedded within healthcare process, which was also highlighted in the literature especially in the context of clinician liability if they do not use AI once such a technology becomes standard of care (35, 47).

#### Digital readiness of the NHS

The tension between trust in the NHS and acknowledgment that in many ways it is not the best suited organisation to develop AI, might stem from poor digital readiness of the NHS. Previous reports highlight poor recognition of digital staff within the NHS and information technology practices that are not conductive to innovations, such as problems with getting state-of-the art software installed, even if it is free (48, 49). Finally, the level of digital maturity in NHS Wales is lower than in similar organisations across Europe (50).

#### Knowledge and attitude towards AI

One phenomenon that is worth a brief discussion is the effect of knowledge on AI attitude. This was an area of contrast between participants. A respondent in the retiree group noted that the discussions during the focus group allowed her to learn more about AI, which caused her to have a more positive attitude towards it. In contrast, MOP2 noted that ‘I think the latest research shows that the more you understand about AI, the less you want to use it’. SFS1 noted that ‘I think I’d rather wait a bit longer until it's like a lot of evidence that has been like basically perfected and we can fully trust it’; yet, this statement is only meaningful if information can change opinions. This has further implications on the extent of information needed to be disclosed under the principle of transparency. If knowledge does not modify attitudes, then a smaller amount of information disclosure could fulfil the obligation to transparency. If information does impact decision-making, then more detail needs to be given to patients, so that they can make these judgments by weighing up various consideration. Some of these responses suggest that knowledge might play a role in moderating attitudes towards AI, which is an important topic for further exploration, especially since respondents in Wales highlighted the centrality of an evidence basis when implementing healthcare AI technologies (8). Yet, some of those with a negative attitude towards healthcare AI stated that nothing would convince them to change their attitude (8). As such, even high-quality evidence might not persuade the most hardened sceptics.

#### Comparison with other studies

A key observation in our study was the importance of human contact during the clinical encounter. This was in agreement with previous work (14), where the patient-clinician relationship facilitated trust and ensured empathy, though our study participants also perceived the involvement clinician as a safety mechanism in case of AI failure. Similarly, in agreement with other work (14), our participant also acknowledged AI co as an enabler to workflow efficiency, and placed a lot of emphasis on data governance. While these topics were not absent, our participants voiced comparatively little concerns about regulatory, financial and leadership issues (12, 13), as well as places not as much emphasis on concerns relating to equity, accessibility, and stakeholder co-design (14).

When compared to other UK-based studies there were both key areas of agreement, as well as novel findings. Respondents in our study were often confused about what was exactly meant by AI and this is a phenomenon that has been highlighted in the past (9). Information governance related concerns were present both in our work and previous work from England (10). Similarly concerns about staff de-skilling were also highlighted in a past study (11). Importantly, our work highlighted that while de-skilling is a risk, participants are aware that changes in healthcare skillset are to be expected as part of progress in healthcare technology and practice. What appeared to be at the core of our participants responses is that due diligence is considered when AI technologies are implemented to avoid harms, which is in-line with our bot survey findings and other UK-based research (8, 11). This is both true in relation data privacy based concerns as well as being expressed by a wish for a human-in-the-loop. Yet, the human presence is not just a fail-safe mechanism for AI, but is also an expression of the fragile intimacy of the healthcare encounter. Our study also raises questions to the assumption that it is the elderly who might be more averse towards AI, while the young might be keener for its adoption. The presence of thematic themes also highlights the limitations of a reductionist approach to healthcare AI assurance, and supports the need for a holistic overview of such technologies during their assessment and implementation. For example, implementing a healthcare AI product developed by a for-profit corporation might not be acceptable to end users, despite the product meeting all the relevant governance and safety criteria, because of wider issues of trust relating to such corporations, as has been already seen in other areas of the digital transformation of the NHS (51, 52).

## Future work

Based on the results of this study, and other work conducted as part of this broader project, policy recommendations will be made regarding ML implementation in Wales. Nevertheless, there are still underexplored aspects of people's opinion on AI adoption in healthcare. For example, in this study we started exploring participant opinions to the use of ML in cases where proxy consent is sought for AI use, but this topic has still not been explored in depth. There are also other practical challenges that need to be overcome, such as how to balance peoples’ requests for giving informed consent to the use of AI in their care when healthcare is delivered in a complex system, where staff are often not aware when AI technologies are used to support them. Moreover, the issue of how knowledge affects patient and staff willingness to use AI technologies should also be further explored. While this study focused on members of the public as potential patients and healthcare staff involved in treatment or therapy decisions (the end-users of the technology), future studies should also explicitly focus on healthcare informaticians and managers to provide a more comprehensive view of the challenges and potential solutions for AI implementation in Wales. Finally, it might be useful to look at the opinions of Welsh language speakers, as well as linguistic minority groups living in Wales, with regards to their perceptions of barriers and facilitators to healthcare AI adoption, as Welsh language promotion is a prominent feature of political debate in Wales, and concerns about chatbots and large language models might be particularly relevant for these groups.

## Conclusion

This work considered the opinions of both healthcare professionals and members of the public, as well as younger and older adults. All groups agreed that preserving the human element of the clinical encounter is vital, and as such AI should be used to improve healthcare processes, and not to replace clinicians with digital applications. This is largely congruent with previous studies in other nations and shows that healthcare AI should be implemented primarily to increase quality of care and not to decrease healthcare costs by replacing clinicians and cutting face-to-face patient-clinical contact. It is also important that AI technologies preserve data privacy, and users might trust such technologies more if they are developed and implemented by trusted organisations that are not driven by profit. Yet, this should not be confused with an unwillingness of patients to have their data used to improve healthcare technologies; rather it should be understood that proper privacy preserving process should be employed to facilitate healthcare improvement while protecting the privacy of patient and organisational data. Moreover, how user data is used should be communicated transparently and not via generic statements, which raises issues over the adequacy of generic privacy statements which organisations might use as part of their AI implementation processes. Finally, when making proxy decisions, participants recognised that it is important to respect the wishes of those for whom such decisions are being made, with the same broad considerations being applied as in other best-interest decision-making cases. Proxy decision-making is not associated with any additional barriers or facilitators when compared to those affecting users when they make decisions for themselves.

## Data Availability

The raw data supporting the conclusions of this article will be made available by the authors, without undue reservation.
