# Supplementary material for: Barriers and facilitators to healthcare artificial intelligence adoption in Wales: findings from focus groups
Source: Front Digit Health. 2026 Oct 12;8:1927799. doi: 10.3389/fdgth.2026.1927799 (PMC13717693; doi:10.3389/fdgth.2026.1927799)
Supplement: Supplementary file 1 [file Supplementaryfile1.docx]

**Guide for semi-structured interviews and focus groups**

Firstly, thank you all very much for taking the time to speak to us today. My name is Michal, and I am a researcher working for CEDAR, an NHS research group, based at Cardiff & Vale University Health Board. I am running this focus group and I am really interested to hear your opinions, so please be as honest and open as possible. Anything we discuss today will be anonymous, meaning that nobody outside of the research team will know that you have taken part. We may use some anonymised quotes in the reports and publications that we write, but nobody will be able to identify you from those.

Before we go any further I thought it would be helpful to go over the background for this piece of work as I know some of you are more familiar with it than others - apologies if I am repeating what you already know. Machine learning is a type of artificial intelligence technique that allows computers to learn patterns from information that is given to them. In Wales, the NHS collects a lot of information using Patient Reported Outcome Measures, known as PROMs, which collect information on patients’ ability to do every-day activities (such as shopping), their comfort and pain, and other ways in which their health conditions affect their life. Using machine learning we could potentially find out which treatments are likely to work best for which patients. We could then use this information to help decide which patients should be offered which treatments or which patients should be prioritised for treatment. But if we are to develop such technologies, it is important that members of the public, patients, healthcare staff and other people involved in healthcare are happy with how we develop such technologies and put them into practice. The aim of this focus group/interview is to gather your opinions on how such technologies should be developed and put into practice.

I’ve had permission from you already, but can you all confirm that you are happy for me to record this session please?

If you want to comment at any time but don’t want to interrupt, you can put your hand up and I will call you in.

Before we start with the questions I thought it would be helpful for everyone to briefly introduce themselves [this might not be necessary if e.g. a focus group is carried out in a group with regular members].

Does anyone have any questions before we start?

1. Could you please tell me what would make you worried about such a technology used in your care/clinical practice? I am particularly interested to know why these particular things worry you. [use data from survey results if necessary]
   - Prompt: Would you trust some institutions in developing such technologies more than others? How would the cooperation between different institutions affect this?
   - Prompt: How would you feel about your /patient data being used for such technologies to self-improve themselves?
   - Prompt: Do you think you/patients should be told when such a technology is used in your/their care?
   - Prompt: Is there any difference/what are the important differences between such AI technologies compared to all sorts of healthcare devices that are used but patients are not told about (e.g. surgical tools or monitoring equipment)?
2. Could you please tell me what would make you more comfortable or encourage you to have such a technology used in your care/clinical practice? I am particularly interested to know why you find these particular things reassuring. [use data from survey results if necessary]

- Prompt: would you be reassured if there was a system to take such AI technologies out of practice/decommission them

3. Do you have any hopes about how the introduction of such a technology could affect your care / the care you provide? [use data from survey results if necessary]
4. Do you have any worries about how the introduction of such a technology would affect your care / the care you provide? [use data from survey results if necessary]
   - Prompt: would you feel more comfortable if you could refuse having such a technology used in your care / in the care of your patients? What would you be willing to give up to be able to refuse the use of such technology (for example, the care process might be slower with out AI)?
   - Would you be reassured if the use of these technologies was periodically reviewed, and technologies that were not beneficial were stopped being used /decommissioned?
5. There may be conflicts between the different things that would make such technologies acceptable to you. How should those creating such technologies and putting them into practice balance these various principles? [use data from survey results if necessary]
   - Prompt: Privacy vs gathering more data to achieve better results
   - Prompt: Clinician input vs speed of decision
   - Prompt: Potential for benefit vs avoiding harm
   - Prompt: Transparency of the artificial intelligence (AI) technology vs benefit it can deliver
   - Prompt: Willingness to answer/deliver extra questionnaires vs potential accuracy of the AI
   - Prompt: Erring on recommending treatment vs erring on not recommending treatment
6. Are there any important differences in what would make you trust such a technology and make it acceptable in the care of yourself or someone who is in your care (such as a child, partner or elderly relative) / in the care of those who give consent themselves or for whom proxy consent is obtained or a best interest decision is made? [use data from survey results if necessary]

Thank you for your input into this discussion and I hope you have found this focus group/interview interesting as well.
