# Supplementary material for: Barriers and facilitators to healthcare artificial intelligence adoption in Wales: findings from focus groups
Source: Front Digit Health. 2026 Oct 12;8:1927799. doi: 10.3389/fdgth.2026.1927799 (PMC13717693; doi:10.3389/fdgth.2026.1927799)
Supplement: Supplementary file 3 [file Supplementaryfile3.docx]

Example quotes for each sub-theme from all four focus groups

**General members of the public**

| Theme | Sub-theme | Example |
| --- | --- | --- |
| Barriers to trust | Black-Box | my biggest worry is that, erm, artificial intelligence, particular the large language models that are prevalent at the moment is that the, the data is, er, not properly understood-, or the, the weights are not properly understood, so the, what the, er, artificial intelligence learns is then kind of intrinsic to the programme and how it performs, but actually scientists don’t really understand, erm, what those weights are doing after they’ve trained on very, very large data sets, so whereas in the past software has been sort of well understood because it’s been programmed and then you put the data in and it’s a data base, er, the Machine Learning techniques, it’s very difficult to tell what it’s actually doing and therefore it’s quite difficult to tell whether you should trust it  if we’re really, really committed to the principles of open, or transparency explainability then black box AI, I know that’s a sort of short term we don’t understand how it gets to its answer, we should never, we just should never go there and if we can’t explain it, so I’m not, not even sure [Name], whether the, it would be more accurate but we don’t know how, that feels like a tautology for me, that feels like, scientifically from my own training I’d say that’s just, that just doesn’t work  I would just come in there and say, and I think, I think you’re right. I mean clearly we are using things that have efficacy, proven efficacy but we perhaps don’t understand why it works. But where’s the liability? […] So this is the questions then about people worry about is like, where’s it liable if, if we can’t explain it |
|  | Foreign ownership | I’d be slightly less worried if those black boxes were being developed, erm, and, and owned within the UK than I would if they were owned in the US |
|  | Commercial ownership | we’ve already seen many, many instances where companies will train their AIs to be very specific in certain areas, be that not able to actually answer your question because it’s political or you’re naming a specific famous person or worse still, giving you answers that are clearly prejudiced, based on that company’s current thinking. |
|  | Data protection | it’s that data protection stuff as well. So, what is to stop a company, once your data goes into that black box, what is to stop them using that data for you personally and looking at, you know, denying people insurance and things like that, erm, because of risk factors, er, so, yeah, a big sort of, erm, privacy problem |
|  | AI not yet fit-for-purpose | I think AI is often just using words as its input, using pictures as its input, perhaps starting to increasing use video as its input but it’s miles adrift from getting that rich input that you get when somebody’s sitting in front of you and interacting with you. |
|  | Lack of human-in-the-loop | if there’s no human in the loop then, you know, frankly, I don’t think we should do that |
|  | Distrust to some organisations | I’m very lucky I have got a specialist hospital that looks after me and they’re very, very, erm, clear in what they do and one, one thing and another. However, my local health board is very different and I don’t have trust with those for various reasons. […] But it’s where your trust is and I wouldn’t like my local health board to use images or any data from what’s happened with me as they haven’t been part of it, if that makes sense and I would have to really trust that professional before I would agree to anything. |
|  | Data bias | the systemic problems so the systemic problems in, in the NHS as well as everywhere else in society means that, erm, for example, er, medical students aren’t trained properly on, erm, assessing various, er, conditions when they are presented on people with black or brown skin, for example and that, erm, er, a lot of, erm, a lot of research on to conditions and medications aren’t tested on women. Erm, so those sort of systemic issues of racism and sexism and kind of built into the NHS at the moment. I imagine that it would be something that if we don’t fix that, that is something that would just end up being, it would end up being, it ended up going on whenever you use Machine Learning or anything like that, it wouldn’t address it, if we hadn’t addressed it in real life first. |
|  | Automation bias | I think it’s going to be very difficult in the future to, and the more people who use AI, the more they get used to thinking, oh it’s just giving me the right answers, I don’t have to look anywhere else, erm, it’s given me 90% of the right answers why should I worry about the other 10%, or why would I even notice that other 10%. Erm, yeah, so I think it’s a really, really large slippery slope we’re on, we’re on the top of.  If you have a black box AI, or you’re not still quite sure how it all works, I think one of the worst case scenarios you can have is that, it’s that it starts producing very positive results. Because if it produces negative results you tend to want to look at it more and scrutinise it more. |
|  | Unpredictable how an AI will work in the future | if you just take paracetamol on its own. Whilst we don’t fully understand how it does the things it does and how it manages to dull that pain that you have, erm, we do know that paracetamol 50 years did this unknown thing in exactly the same way as it’s doing it today. That’s the massive difference, that if paracetamol was an AI, you’re most likely to find in five years’ time, not only is it doing your pain management, oh, but it’s also doing this. We’re not quite sure why but hey, it’s a good thing, so we’ll just leave it be.  […]  No, I think it was just, it wasn’t a great analogy because with paracetamol, whilst it’s an unknown it’s a fixed unknown. AI is never truly a fixed unknown, it’s always, it will always be changing. Erm, so I think from that point it’s not necessarily the same in terms of well we’re not sure how it works, but it’s okay to use because the outcomes are always positive, erm, and as I said with paracetamol yeah, we still don’t fully understand how it works but we know it does work and we’ve had decades of it working, but it always works the same way. It never changes.  […]  But then, then the outcome from that algorithm maybe fed into other ones and […] this is the problem, where you build up a network of them, erm, and as, as [Name] says, as [Name] says, it’s the weights that is used by those, those different outputs that we don’t fully understand why it applies them in certain aspects and not others. |
|  | Complicated digital environment | I was consenting on his behalf I’d be like, fine, if it’s right, you’re using AI to look at images, you’re using AI for your work process but as soon as it’s oh, he will now need to have an app, he’ll need to have a smartphone. He’ll need to phone a robot and talk to a robot in a call centre somewhere to give them the information if he doesn’t have the app. Instantly it would be a no and I’m someone who wants AI to be embedded into health care. |
|  | Difficult to test AI in-situ | with AI, it, it’s a completely different kettle of fish, as, as both [Name]’s have said, erm, the minute it evolves or changes… Seriously, the amount of test, retesting you should do would probably be too much. It would then hinder the AI in being able to move forward because by the time you’ve tested this evolvement or this patch |
|  | Changing the way we work | It’s just how you get to that point but I think being able to show alignment with similar pieces of work, similar automation, similar ways of, we don’t operate the NHS like we did back when it was created and there’s reason why. When my mother had a caesarean she was in hospital for a week. These days they bowl you out, you know, the next day, sometimes the same day if you’ve done really well. Things have changed, we’ve got to kind of evidence those to show why this is the next step for us.  just a couple of points, deskilling, I think we just need to change, not deskill, just change. It’s not deskill-, if we use the deskilling argument then basically we’re never going to do anything. Erm, and this goes back to any introduction of any technology in any regard, whatever you call it. So, we’ve just got to train better and learn better and change, not think about it as deskilling. Erm, the fact that we can process more pathology tests faster, give diagnosis faster, that’s not deskilling, that’s actually helping the pathologist do their, cope with the volume of work that they’re doing, it’s not deskilling. It changes the way they work, but it shouldn’t be about de-, deskilling. |
|  | Data for continuity of care | I’ve got personal experience, mother nearly died because data wasn’t shared between departments because each department’s got their own little system or their managing their data and they’re not sharing it. So, the sort of basic levels of use of technology to drive that benefit that we all want is quite low.  am I bothered about continuity of care if the data that the, the GP, practice nurse, physician, assistant has got available to it is comprehensive. If it’s comprehensive, if I get the right, if I get the outcome in a timely manner, do I care the fact that I’m, you know having to talk to different people if they have got good recorded data about my healthcare or care plan depending on what, what it might involve. So the fact that I don’t see my GP is, erm, I’m a bit less bothered about that, but I am bothered if they didn’t record the conversation properly and haven’t perhaps asked some broader questions that might be relevant and, because they didn’t have time to do that, |
| Concerns about the NHS developing AI | Lack of resources | it would require a lot of funding and I, I think that as always is going to be the biggest hurdle […] building it in house would be great but I just don’t see we have the kind of resources to do that |
|  | Not the role of the NHS | building it in-house would be great but I just don’t see we have the kind of resources to do that and, you, you’re moving away from the what the NHS is really supposed to be about I, I would say in that respect. |
|  | NHS not great at innovation | We need innovation to actually happen. It’s not going to happen inside the NHS. |
|  | NHS digital weakness | I think the public probably don’t understand how poor the NHS is currently in terms of it’s use of technology and data . Forget about AI, the level of integration, the level of knowledge the level of failure through to manual processes that doctors and nurses have to deal with and then the public have to deal with is, is, is not understood. |
|  | Need for private organisation input | It’s not going to happen inside the NHS. With all the will in the world, we do need to encourage, erm, organisations of all sizes and there are lots of amazing organisations in the UK that are doing work in this space but we need, we really need that. So, I think we just need to manage the rhetoric a little bit about commercialisation of the NHS because frankly, if I get my diagnosis quickly, I see a doctor quickly, I get my treatment quickly, Do I care? And I wonder whether sometimes we’re a bit stuck with the rhetoric of the NHS and not stuck with the outcomes that we want. |
| Understanding how things work | AI is not one thing | I think the most important one that we all fall into the trap of is thinking AI is one thing and it’s actually many different things. A large language model as [Name] has indicated is very different to Machine Learning, predictive analytics or image analysis. These are very different things. They all get badged AI, they are AI, but they are different things and they have different risk profiles and different understanding and I think that’s, that’s important then, when we come to communicating the benefits or disbenefits of these, er, products as they enter the market and as they’re used by the NHS because if we just say it’s AI, and then we all get very worried about it because it’s, you know, it's got a sort of sense of science fiction about it, we are really confusing the issue. |
|  | NHS staff not understanding digital | I’m old enough to remember the first home computers, home computers, erm, I learnt to programme, because you had to programme. It wouldn’t do anything otherwise. I’ve got a good understanding of what computers do. We had an old phrase which a lot of you probably know, garbage in, garbage out. What you put in, is only as good as you get out. What you get out is only as good as what you put in. My children brought up, mobile phones, tablets, they have no idea how they work or the sort of, erm, concepts behind them and I imagine there are all the young medical students now in their first, second years have got a similar level of understanding so I don’t expect that they would, and nursing students they, they don’t necessarily understand how these things work so, for them to be able to explain it to other people and explain the issues behind it, or even to know about the consequences, they’re going to need that added to their curriculum. They’re going to need thorough training on things like that so that they can understand how to use it. I, I don’t think it’s going to save any money.  what would really make the difference is that if that, if primary care staff genuinely understood what, what it means and how it works and the difference between, erm, population data and how that relates to the individual |
|  | NHS shortcomings | I, I’m sort of constantly amazed how people worry about new technology in this context but yet have no information about the level of failure that’s currently operating in the world of the NHS today. I mean in some respects we’ve had some big scandals about, er, blood, er, blood donorship, erm, there was another one yesterday about cancer, er, involved with, di-, er, pregnant women, I think yesterday, if I heard the news right. And we, we don’t know how many times, how much failure is in the system because we, we don’t provide the right information to the clinicians to make the decision at the right time or actually. It’s covered up that they missed a diagnosis because they’re only human, and, when you’re analysing hundreds of pathology slides, trying to understand where a cancer diagnosis might be, course you’re going to make mistakes. We, we’re only human but because we don’t communicate that, we then get a bit stuck with new technology and that’s going to be really problem-problematic. |
|  | Knowledge as a disincentive to use | I think the latest research shows that the more you understand about AI, the less you want to use it |
| Creating trust | Public engagement | I think as long as you’re honest and transparent about it, so people know up front that AI is being used and that the people that they are speaking to can adequately explain how it’s being used. It’s not enough to just have a vague disclaimer saying, you know AI may be used to process your data. If you then, turn round as a patient and say, right how is AI being used in my care, you can then say, right, it’s going to be used to look at this lung scan. It’s going to be used to look at this scan. It’ll then go through to, erm, a doctor who will review it, or it will flash, people are fine with that, but when it’s just a vague disclaimer, |
|  | Explaining key principles of good AI | I think the really, the really import-, important point is that principles, being really clear what they mean and sticking by them. Things like human in the loop, explainability, privacy, security. Engage the public on that, share very openly and transparently what the current position is and then, er, and then take, take it from there |
|  | Familiarity breeds comfort | So, I’m just thinking of 15 years ago when, if you wanted your blood pressure taken you had to go to your GP for your appointment and you would use an entire GP appointment just to get a blood pressure reading. Then they bought in the machines and you could do it yourself and lots of people didn’t like doing it themselves because they were like, oh, it might not be right. I want to see someone. I want to speak to a person, but gradually people accepted them, behaviour changed, people got used to giving the receptionist the piece of paper and now our systems have got to the point where that data gets input. I don’t know if it’s input manually or if it’s bought up automatically, but it now flags up if there’s an issue for, if that person is outside of a standard norm, but also if they’re outside of the norm for them as an individual, based on their previous readings and then they do get that face to face contact. So, we’ve got to a point where the people have accepted automation. |
|  | Put controls and gather data | I think if you’d said this is a focus group to talk about digital imaging and how that can improve services, so all of your, yeah, all of your lung images go through and it gets spotted by a machine, that, that’s quite clearly, your tests are going to get seen by somebody, even if it’s a machine and that, that’s quite clearly got data to back it up. It, it works. Erm, obviously there’s a margin of error and then you, you always need a person involved, but, you know, I wouldn’t have a problem with that.  I wanted to draw the analogy with approval of drugs, so the question is, you know, you’re producing quite a complex tool that is going to be used in some way, and it may be used in clinical decision making and in treatment, and the question I’d have is once you’ve developed this thing, and you’ve trained it, and all this, er, knowledge is embodied in it, how do you then validate that it’s working in, in a defined way, what process does it go through to make sure it’s performance is well understood and it’s limitations are understood and do you generate something like, you know, NICE guidelines, erm on how it should and should not be used and then building on that, I would say that if the AI or machine learning is retrained you’ve practically reformulated a drug and you should go back through that process again. |
|  | Don’t complicate patient experience | The patient experience should be being improved by it, it should be more seamless it should be easier, it should be quicker. They shouldn’t be asking them to do anything extra in order to utilise it. |
|  | Trusting some clinicians | obviously with surgeons they are, erm, specialists and, and as I said I go to […], they, they train, they really do train to the, the best highest level. So that’s’ why I was saying in my comments where if one of the surgeons came to me and say that I needed a new knee, you know, sometimes soon, and he wanted to perform some surgery which will actually have AI, I would be quite happy to be a guinea pig for that because I, I have full trust in, in the hospital and you know, the, the aftercare, as well the surgeon, erm, that would help him to be able to continue. |
|  | Ensure face-to-face time | But with surgery it’s a different look, which I don’t know how we’re going to change that, because it’s a ten minute slow and you’re out before you’ve even sat down and it’s, it’s as if there’s no compassion any more.  […]  it would be nice if we could actually change that, where the GPs have more time with the patient instead of, you know, like I said, that ten minute thing and, you know, if you’re a complex case with other, erm, you know, problems, they’re not going to be met,  I wouldn’t want it to say, to take away from face to face time, because having spoken to my relatives who are a GP and a physiotherapist, they get so much out of just the informal conversation with the patient and the way that feels, understanding their soc-social situation, understanding what the local, erm, sort of culture is if you like or, you know, socioeconomic position, erm, they may pick up on things that aren’t even what the patient is raising because they can see it in front of them, they’ve got so much experience.  Yeah, and continuity of care I think is another big one as well because, you know, my GP surgery, certainly doesn’t have continuity of care it just assigns to you whichever GP is available. Although there is a named one, I’ve never seen him, erm, so continuity of care seems to have gone, whereas talking to me, erm, now retired GP relative, she knew all of her 2000 patients and the names of their pets, you know, it’s a completely different, er, environment really. So I think that face to face time, continuity, understanding the person and their family is, is something that we’ve lost somewhat and |
|  | Expert understanding | Yes, and I’d feel much more that I trusted the AI if I knew that there were people who did understand how it was working |
|  | Staff training | It won’t necessarily save any time because there’s a whole lot of, erm, work for the people who are going to be using it, to understand how to use it. I see it in work. People have no clue how to use systems so, erm, yeah  that is going to go into GP surgeries and all we need to now, is, is trust that the, erm, the GPs are going to have training in that. |
| Supporting the healthcare system | Data used for public benefit | I think if data appropriate anonymised as in they don’t know it’s [Name] but they can have a look at my lungs because it’s really important for overall population health to reduce demand in the system is absolutely vital if we are going to stop going into bloody hospitals and start, er, having the ability to sort of look after ourselves in different ways, so I think that, that’s really important that we, we see ourselves as being part of that system, appropriately anonymised and that respect for privacy and stuff like that. The data protection rules I think we’re talking about. So, again, I’d just sort of give a sort of perspective on that, about how we manage demand because we can’t keep shoving more in, we’ve got to take a little bit more, but we need to learn more and our data is absolutely vital for that learning.  I don’t mind my data being used to improve the model, so if I have a prostate biopsy and the, the data’s used to help my diagnosis but then it’s anonymised, it can then form part of a broader cohort to improve the quality of the model, I think that’s very valuable. People are very, once, once they acknowledge and engage properly I think people see, have got very strong altruistic, er, perspective on that would want to see, er, it improve because it comes back to this over-, overall population in health. |
|  | AI can support tasks | but it’s a guessing game, like a, you know, erm, a post code lottery where those children can be helped because the longer that they wait for that surgery, the delay on their education, because if they can’t hear, it stops them from, you know, reading and writing.  I am bothered if they didn’t record the conversation properly and haven’t perhaps asked some broader questions that might be relevant and, because they didn’t have time to do that, then I’m, then I’m probably more bothered about continuity of care and this is where AI actually could help. because what’s something called ambient transcription, the ability to listen and record, which you’re using today [Name], in this conversation, record the conversation and transcribe it gives you the opportunity to engage directly with us and listen empathetically which you, you’re doing, obviously. |
|  | Individualising care | you can get a computer to do it, it’s not a big deal and more roles in the health service for more of that kind of like patient support, really practical patient support, in person where they can actually talk to a person |
|  | Automation of tasks | If you had something were AI could just automatically do that on all the systems where you can pick up that right, this has changed. It’s such a minor change but for the patient it makes this so much easier and from an admin burden it makes things easier and there’s no significant risk there  it’s what AI are we using, how are we using it, and it should being used to relieve our working pressure, whether that’s clinical or administrative without the patient really needing to see that it’s changing anything. |
|  | Avoid clinicians missing signs | I have said that in my message that, that I, I do believe that, erm, even though I had an MRI scan which is supposed to be advanced, erm, they only look at one […], and presumed the same in the other. So, they thought it was septic […] in one […] and […] other was the same instead of actually going into identify. Where, obviously AI if, if they knew about sepsis or whatever that would actually flag up and as I’m learning now because I want to know more to help others, erm, AI, AI could help, er, sepsis, you know, erm, patients in the long run if it can identify earlier. |
|  | Opportunity for learning | knowledge is built into a very complex set of, er, weights, that are, you know, and we know algorithmically how they’re applied, we could write the mathematical formula but we’ve got no idea what they mean. Erm, and I think that is an opportunity actually, as well as a threat because I know there’s research going on to better understand how, er, programmes like Chat, ChatGPT are interpreting the world, and there could actually be research directions where you just go into those algorithms, understand how they’re making their decisions and that might actually give you a new way of thinking clinically. Erm, so I think there’s, there’s a real opportunity over the next small number of years to say, don’t just do machine learning and accept the results but dive into the detail, dive into the weights, and understand what discriminations that algorithm is making that give it the, the success that it’s got because those underlying concepts could be really key. |
|  | Supporting healthcare through technology | So, I’m just thinking of 15 years ago when, if you wanted your blood pressure taken you had to go to your GP for your appointment and you would use an entire GP appointment just to get a blood pressure reading. Then they bought in the machines and you could do it yourself and lots of people didn’t like doing it themselves because they were like, oh, it might not be right. I want to see someone. I want to speak to a person, but gradually people accepted them, behaviour changed, people got used to giving the receptionist the piece of paper and now our systems have got to the point where that data gets input. I don’t know if it’s input manually or if it’s bought up automatically, but it now flags up if there’s an issue for, if that person is outside of a standard norm, but also if they’re outside of the norm for them as an individual, based on their previous readings and then they do get that face to face contact. So, we’ve got to a point where the people have accepted automation. |
| Consent to AI | Right to refuse AI use may not be practical | In terms of revoking the right to use data I think, you know, I would love to say we should absolutely be able to say you cannot use AI in my treatment at all because it is your right to decide how you want your treatment dealt with, but then I think, well, you wouldn’t turn around and say, oh no, I, I don’t want to see this particular professional if that is the professional who needs to deal with you. So, if, you, you wouldn’t say, oh I don’t want my thing to be sent via Outlook or I don’t want this record being shared with other members of the clinical team. So, I think there is something about where we have to encourage patient trust, that if that data is put through AI, there is a reason behind it |
|  | Same standard for self as others | So, I would tend to use my viewpoints for other people as well just on the grounds that, just on the grounds that if they can’t, erm, er, give their own consent, that I want to be, erm, more careful but it, it would be the same issues that I would be worried about for me, as for somebody else. |
|  | Respecting other’s wishes | But, if they, if I was an advocate and they did not want to consent then I, I’d obviously have to respect that. |

**Healthcare professionals**

| Theme | Sub-theme | Example |
| --- | --- | --- |
| Confidentiality | data | the biggest things I’ve got at the moment is around confidentiality putting sort of, for example, patient or business sensitive information in AI tools |
|  | external servers | I’m aware it probably goes off to external servers and things and you, you just worry about the safety of any information that we put into it  I think there are areas where it can be used which is relatively safe because I think the patient data remains in the same organisation |
|  | information security | then I think I worry about data leaks, reputational damage and personal liability versus health board maybe. Erm, you know, probably both, both of those  So you can actually use commercially available model to analyse images but then only, only you would know which patient it is and the commercial software will never have access to, er, patient dat-, patient identifiable information. So, only your NHS model will, will know who is who |
|  | even trusted systems fail | it doesn’t necessarily mean that I’m, I’m convinced fully because we, we unfortunately know of experiences where data has been breached in the past when systems are meant to be in place |
| Governance | lack of guidance | we do have something in the health board and, but it’s come without any sort of training, learning, limited guidance tools, so yeah, that’s where I feel. I think we’re all quite wary of that generally, or quite aware of it in healthcare, yet this has sort of come with limited guidance |
|  | lack of training | we do have something in the health board and, but it’s come without any sort of training, learning, limited guidance tools, so yeah, that’s where I feel. I think we’re all quite wary of that generally, or quite aware of it in healthcare, yet this has sort of come with limited guidance |
|  | need for standards of behaviour | so then there’s, there’s still the high level of requirement for the human to, to play their part correctly which still then comes down to, well what are the standards by which we’re working and behaving and acting |
|  | regulations don’t stop bad practice | without this coming across too cynically, that doesn’t stop malpractice at the moment and things, so, you know, again, that’s a bit too extreme but it doesn’t stop a varied practice shall we say? |
|  | AI decision-making subject to the usual legislation/practice | all the rules apply whether you use AI or not and so therefore your decision making shouldn’t be any different. |
|  | need for governance | again not without the governance and the legislation of it |
|  | consent from patient not needed if HCP is the final decision-maker | in terms of consent from the patients, if we used it as a tool, purely like we do anything else, you know, using, er, colleagues advice, using the AI to generate information so, we would be the final decision maker and the final one making that clinical judgement then I question whether we would need consent  if it’s facilitating a decision and you’re, as a clinician, taking that decision, that’s fine and we probably, you, you know, you probably wouldn’t necessarily cite all the sources we’ve used |
|  | lots of private software in the NHS so what would NHS owned AI change | we already use private software for our rota, the labs that got hacked in London used private software, the quality and safety has private software so already there are companies, and actually the data that we submit for ICNARC is done via a private software company, so to now say that AI is going to be NHS owned but we use a lot of software from commercial companies, I don’t see how that’s going to work. |
| Technical understanding | how it functions | I think it’s where it comes from a sort of lack of understanding and knowledge at present of, of it, so it’s quite hard to articulate what, what drives that concern. It is, it is just that, yeah, that lack of knowledge and understanding I think |
|  | how it is developed | I guess my first thing about AI is I didn’t understand what databases have been used to teach AI. So the question is, have they had access to journals which are behind paywalls, to be able to have the better knowledge available to be able to make a decision. |
|  | it is not just about who owns the AI but about how it functions | I mean, ownership and money is one thing. It’s employing the vast majority of the population and giving them a purpose to get up in the morning, is the other, isn’t it?  if AI was, erm, managed, controlled, delivered by a, er, a govern-government organisation and not for profit and things like that, I think I’m, I’d have a personal bias that I probably would be more favourable to that. I think, erm, what I’d have to admit to myself is that unfortunately that doesn’t mean that they’re the cutting of, of those industries and that’s, erm, that’s the sort of give and take that you have with these things. |
|  | can AI deal better with comorbidities | what we have is an increasingly comorbid population and so what I, what you also find is, is if at the beginning you go down the wrong route, so, if at the beginning somebody puts you on the wrong guideline it takes two or three days for a diagnosis to change and I guess what you might be saying is AI’s going to make the right diagnosis. I mean it might and put you on the right pathway, but very often people have a combination of problems, not necessarily just one. |
|  | do many private systems present less of a hacker target | if we did have that [an NHS full patient record system] it would be such a sort of, hackers would be just constantly trying to get into it and take it down I think. It would sort of target number one. So, I think in a similar way if we had NHS AI that, again would be such a sort of, you know, attraction to hackers rather than maybe having it, being a, you know, being in private companies and perhaps a bit more disseminated. |
|  | rare diseases | I’ve used AI, for rarer diseases it is not that good.  I think in histopathology, I think progress has been massive and that actually, er, my, my understanding is that AI could be better than most people in rarer diseases with histopathology |
| Worries about consequences | reputation | then I think I worry about data leaks, reputational damage and personal liability versus health board maybe. Erm, you know, probably both, both of those |
|  | liability | then I think I worry about data leaks, reputational damage and personal liability versus health board maybe. Erm, you know, probably both, both of those |
|  | outcomes of decisions | my point was, was to go with the fairly soort of obviously of, erm, worry around clin-clinical decision making, er, the risk that, that potentially comes if we, we rely on sort of machine learning to make these decisions. Erm, and the, the, inevitable accountability and where that lies. |
|  | cannot blame AI or reject its recommendations | My understanding with the introduction of AI is that the clinical governance and the risk is taken on by an organisation and that the organisation purchases the AI software and therefore takes responsibility to a certain extent and then I share [Name] concerns is to me, I guess as a doctor I feel that ultimately they want a human to be responsible so, if AI percentage wise is correct more often than the clinician, then the clinician has to justify why they don’t’ go along with AI and to me that feels like you can’t blame AI, you have to blame an individual and I, that’s my perception. |
|  | AI bias | it’s recommended a particular brand, recommended a particular model. Erm, it’s not always the best model and it sometimes been, erm, kind of pushed, it feels like AI is kind of saying this is the better one and from clinical experience I know that it is the better one, for the first six months, but then it historically breaks whereas other models don’t. So I guess my worry is, is the AI systems are trained and on biased data and whereas I think a human, erm, can understand and reflect on the mistakes that it’s made and will then change the way that it, well we change our behaviours and if we change our kind of approach |
|  | deskilling | my only worry would be using, becoming too dependent on it and losing my ability to critically think and critically judge situations and, and that’s priceless really from experience of, you know, seeing patients with different, erm, social backgrounds, cultural background |
|  | not suitable for safety-critical tasks | but anything, any safety critical task, erm, I will be less critical, er, less safe, less kind of confident with, erm, delegating someone’s like responsibility for someone’s life. |
|  | unemployment | the problem is that it becomes more efficient, it saves money but then you lose jobs and I… I guess I worry that this is the same thing industrial revolution we will have large amounts of people who will lose their jobs  I think what usually happens is people find another profession, erm, and, erm, what I think would happen is basically, erm, people who will be losing their jobs because of AI, they will be just finding another, erm, task to do and other things to do in other profession, but will need to, erm, acquire, I don’t think it’ll be painless. I think it will be a lot of pain, er, during this process. |
|  | power of AI corporations | whether there will then be a small number of people who control or who own the AI software who will make all the money and that the difference between those with high income and low income will become massive |
|  | time required for initial set-up | that initial offset of, you know, writing guidelines, policies on how to use it, getting them approved, you know, getting staff, reading the policies, mandatory training all those things in the beginning |
|  | issues in recruitment and training | We’ve been thinking about it a lot because we, we’ve been, had some doubts, with some candidates whether they use AI, erm, so it almost feels like we need to go back to oral interviews and to see people in person, erm, and to, but it’s not a silver bullet |
|  | using UK data vs world-wide data | if it was sort of tried to be run by, contained by the NHS for instance, is there a risk that we’re also closing the data pool that has access to, er, you know, and it becomes like a just UK patients and we’re basing decisions on that data rather than people across the whole world. |
| Creating trust | providing education about technology | they went to some lengths to explain that the encryption was, was to the standards that we, we would hope and expect, and that reassures me to some extent |
|  | preserving the ‘human’ element in decision-making | but then the decision making and the conversations and the treatment possibly within dietetics anyway, we would make that final decision with the patient |
|  | using AI as part of a ‘blended’ approach | I think I would naturally lean towards you know a blended approach […], so, yeah, that idea that it is a part of the, the information, a part of the MDT, erm, a consideration to that, allowing the skills of the, the healthcare professionals […], to bring in the, the human side, the context, the sort of, erm, personalised aspect of things as well |
|  | preference for familiar sources | The honest answer is, I mean you know, I think we all look at AI, we all just have a quick see what it says. It is if I have to choose between AI and, and UpToDate, I would choose UpToDate.  why do we have to acknowledge AI, did anybody acknowledge looking at Google, did you acknowledge looking at papers, it’s because it’s new isn’t it, because it’s new, people don’t know what it is, so then you feel you have to acknowledge it. In five years’ time, you might acknowledge it now, in five years’ time nobody will be acknowledging it, because it’ll be the norm, |
|  | AI takes specific/lower risk tasks | it would be a closed model and that clinicians and health care professionals could only use it to a certain level maybe, because some people might become more reliant than others so, so if you were only able to put a certain amount of data in, and it would support you with making that final decision |
|  | using local models | you can treat it as a black box and basically control what goes in, what goes out and you can have the model which is basically your local model, and you can control what’s going on with it and it doesn’t have an access to the external world, you can keep it inside your department |
|  | who develops the AI | if AI was, erm, managed, controlled, delivered by a, er, a govern-government organisation and not for profit and things like that, I think I’m, I’d have a personal bias that I probably would be more favourable to that. I think, erm, what I’d have to admit to myself is that unfortunately that doesn’t mean that they’re the cutting of, of those industries and that’s, erm, that’s the sort of give and take that you have with these things.  we can use all the benefits of these new, erm, models you’d develop with private companies which might be developing them pretty quickly and, but the NHS we can have a more conservative approach and probably move a little bit slower, erm, but then the advantages that we can have our own model which is, erm, with an access to patient information but there’s low risk of leaking it out.  NHS or the government have kind of a say in, in the development of AI and I, I would almost lean more against that. I think it would be better for private companies to have access. I know that, erm, then creates other problems that we’ve kind of spoken about but I think I, I think AI would grow and develop much, erm, at much quicker rates if it wasn’t owned by a government, erm, department. I think it would be a lot more streamlined and quicker if it was privately owned and |
|  | AI being able to focus on specific conditions | if we were seeing someone with inflammatory bowel disease, we were able to see what the, the AI has learnt from every single patient scenario that it’s had inputted over six months and, as a clinician you, you’d be able to see, wow, this worked for this patient. Would it have any ability to be able to formulate that data and give you an evaluation on saying right, okay, out of all of these patients that we’ve had inputted this is what’s worked best. I just think that would be really useful if you could section it off into different conditions. |
|  | learning AI on your population | Mm, yeah, and put, put the outcomes in, you know, if there’s a way to put those outcomes in with each patient, follow ups, all that data to go in, and then evaluate a bit like a, a journal really that you’ll be able to evaluate the outcomes. |
|  | federated learning | I guess one of the solutions might be having a proper decentralisation, so to avoid having a centralised database, like a honey pot which will be attracting all the hackers, […] so one of the solutions could be that each trust and board keeps their identifiable information on their own services, without internet access, er, but all the raw information, all the anonymised information then can be shared with anybody |
|  | quality assurance | I think we, we need to do quality assurance on it, we need to be doing checks and, and that will always be there. Erm, the same as checking someone else’s work, when we prepare a plan of treatment one person does it, the second one checks, so there will always be someone checking it’s work |
| AI benefits | can be more accurate than humans | my point is not to be naïve to, to know that there is, there is scope for artificial intelligence to be more accurate than humans in some degree |
|  | not implementing it as a lost opportunity | I think we really cannot stop this train. Erm, it, it’s going, it will be implemented everywhere and my worries also being behind that we might miss another opportunity to innovate and that’s, I think, another risk because I remember some of my professors were, were thinking using internet is cheating, er, and now like, look, what’s going on now. |
|  | save resources | We’ve already got the software for it, erm, so it’s actually instead of, so we’re not that worried about AI implementation […] but AI is working like your assistant to automate things, to speed things, to save the costs |
|  | can counterweight human bias | Because I think as clinicians we’ve got our own biases, we’ve talked a bit about bias, and you know, as humans, a clinicians we’re not free of bias… But, you know, there’s the ones we can recognise, there might be subconscious ones, so this, an AI facilitated decision or treatment plan, for you to table that alongside everything else and then, as part of a best interest decision, I think it could just give a different perspective, different angle. |
|  | automation of simple tasks | everything I could really, everything boring, everything which needs automated, automation, erm, anything where I can make a mistake because I’m tired, erm, because it’s, it’s probably more reliable for things like that. |
|  | potential to help with complex tasks | but actually it’s getting extremely useful in complex tasks. |
|  | good at understanding large amount of data | there is something that is, is better equipped at, a, erm, calibrating these trillion data points than I am, than our group is, and things like that, and so those, those are the things where I’m like, well clearly there’s, there’s gains and positives that should come from that |
|  | more time to focus on complex tasks | I would be very comfortable with delegating boring work to it and automating everything I could and saving time for something complex, where human judgement would be needed. |
|  | expand service provision | community outpatients have quite tight referral criteria, I think a lot of services do. So, then there’s a lot of people that want to access the service and they, they don’t meet the criteria, yet still probably have some sort of need or, I think the classic for dieticians is every patient comes to see us and they want a diet plan, they want a meal plan and we don’t really, you know, don’t al-, don’t always do that, erm, but actually AI can do that in seconds and give something really tailored to the foods they like based on their conditions. Erm, so I think it’s maybe that sort of facilitative tool that could be doing things that we don’t routinely do |
|  | focus on patient | I think in the interest of patients, if I was a patient, I would probably like, with probably want, erm, er, my, my doctor to basically focus on, yeah, on making my treatment more person-, more person orientated, to make it better to, to improve things rather than filling in boring reports, sending emails |
|  | fast AI advance – risks and opportunities | I think in 10 years’ time it’ll be basically smarter than, than any human being, er, probably and, erm, I think there’s no, erm, I, I’m being quite hopeful about it, I think we’re, each of us will get a super useful assistants, that will be helping us with every day work, erm, so, yeah, there are risks as we discussed but there are, er, great opportunities I think for innovation |
| Public services not digitally ready | poor digital safety systems | we know they are recurring viral attacks, sophisticated systems who have what I would consider to be up to date software of which I don’t consider the NHS to be on the forefront of that |
|  | paper records | My understanding with AI is that it needs accurate detailed information and to me, we’re starting on the wrong end, yeah. We’ve got paper records, as if you want to have AI to help you with decision making then basically you need an electronic system […] we need the basics first before we get AI to help us with any clinical decision making. |
|  | does not attract greatest talent | I think as well it would, erm, be appealing to those who are brilliant in those areas to develop that. I think private companies can pay more so they’ll attract people who are the best of the best, and then develop these AI softwares, whereas I think government departments, erm, typically don’t pay as much as private companies, so they wouldn’t attract, erm, the best of the best |
|  | do we need AI or just software | my understanding is that the NHS could save billions er, through admin tasks being automated, whether that just requires software or AI I don’t know the answer to that but if you look at some of the things that we do in management it’s ridiculous |

**Sixth form students**

| Theme | Sub-theme | Example |
| --- | --- | --- |
| Errors | AI errors | I think it’s worse if the machine makes an error just because you, you do expect AI to be all knowing sort of and if it does get something wrong it’s kind of like, it could be wrong for so many different things there. Erm, and being, like a machine that’s meant to get everything right, being wrong in medicine is like a big deal, it could be risking lives and things. |
|  | Human errors | there’s always the risk of human error, but then there’s always the risk of the AI going wrong.  Just because it’s the patient telling the AI what’s wrong. I, I, I don’t think, if, if the AI knew everything about the patient, and the problem, I think there’s no problem. I think it would be fine but it’s just the patient isn’t as knowledgeable… |
|  | Humans more reactive to errors | I think humans have more of the chance to take responsibility for it, compared to the machines. So, even if both make, both a human and a machine makes an error it’s more, more likely that a human can take charge and they can get compensate for that error, in a way, like try to make, make up for it all, whereas a machine it might be way worse. |
|  | AI as part of the MDT | Erm, and they’re so used to that, that that’s their kind of, er, default but yeah, I think it’s, the AI work alongside the healthcare professionals and so it’s like improving it sort of thing so that the, the trust that the patient has in the doctor is kind of going beyond the doctor and do AI software that they trust as well.  I would like to say, is that, when we say that doctors are diagnosing, it’s not just one doctor by itself, I think most doctors they go to other like people, to like say, do you think this is like a fair diagnosis which is where an AI might be limited in that case. Like sure, they may have multiple, well yeah, they would have like multiple data off like other people as well, that might be… Mm.  But it’s interesting, it’s a very interesting idea. Yeah, I would be for sure, let’s try this out. |
|  | Automation bias | I just think that the AI being there will, the doctors will just rely on it, just because I think as humans we like to make things easier for ourselves, and if that AI is there telling me everything, you know, about every patient that has ever been treated in the, under the NHS, and it’s just giving me a nice summarised paragraph, erm, and you know, the most likely diagnosis. I think I’d just use it, because it’s right in front of me and, you know, it’s backed up by so much data and then okay all of those people were there, you know, discussing it, but that information’s just right there. Erm, So I think, yeah…  I think, I don’t know, I think with-without the presence of AI, there’s more of that sense that you need to figure it out yourself, erm, and, or with the help of someone else and then, then you consult with other people, you know? I just think that if, in that process of trying to figure it out, there’s this AI that’s figured it out in seconds, you’re going to take that route, erm… |
|  | Openness about risks | I don’t think I’d mind as much, but I’d still ask for, you know, what are the complications around it, what, you know, what are the risks |
| Governance | Transparency | I think like patients, they don’t have, their issue doesn’t lie with their data being used for improvements but I think it’s the, the lack of transparency over the matter that they’re not, because it’s quite a new, devel-, like it’s very apparent in this day and age but this, in some aspects is very new so I think there’s quite a lack of knowledge on the technology and how, how people use it to improve technology in health care, so I don’t think, I think people want you to use their data if it means making an improvement that’s’ going to benefit other patients in, in the long term but I think they don’t really know how that works, so I think a bit more honest and transparent on how organisations do use that data would make patients a little bit more comfortable.  if a patient has a particular condition and the healthcare professional outlines how it would, how it would take a particular approach into helping them with their condition, than a patient would take it a little more interesting and be more invested in it.  Yeah, I think if the NHS were like, at least initially were releasing like two reports every year on, you know, the success of it, erm, and, you know, I think as the years went by and, you know if it was proving successful I don’t, I think like the world would trust it more. It’s just that initially I think that people have issues with it, like with, in terms of comfort and… |
|  | Confidentiality | Confidentiality. |
|  | Exceptions for research | But that also depends on what kind of, if it’s like a research and then I guess like some sort of, oh, what’s the word, er, deception is okay, just to see that it is actually the AI or the technology that is improving their care, not anything else that they do. |
|  | Disclosure of AI use | Yeah, I don’t think it’s right to, you know, allow a patient to go in unknowing especially because it is health care and it’s so important.  Probably not, er, from personal experience, a family member of mine had like surgery and we were lucky that another family member was like a health care worker so I feel like most of the knowledge that family member had was from the person who was a health care worker rather than talk to the other people, because they can’t go into like the technical things just like, this is what’s going to happen, this is all you need to know, if you have any questions, ask.  […]  Maybe at the start, like this is what they’re planning to do but obviously during surgery that’s not possible.  I think when, when you go in for like a surgery, the surgeon doesn’t say, oh, we’re going to open you up with a scalpel, we’re going to use this instrument because it’s kind of like, er, the expectation and what the patient assumes is going to happen. Erm, but I think if you’re putting that, if you’re aligning that with the, the intervention of AI health care they’re not, it’s not, it's quite new, they’re not on par with each other so it’s not, I would say it’s an expectation but if there is a particular development, erm, that does advanced care, it would be obviously a breakthrough but it wouldn’t be an expectation as such, next to like a, a standard of care.  […]  Then it would align with how we have our, our standard treatment now, so I wouldn’t have thought so. |
|  | Trust in healthcare professionals | There might be one little thing that they’ve noticed and they, you know, tell the AI and the AI’s are going there’s nothing wrong, but then, you know, a doctor would do all their like tests or whatever and see that oh, there’s a serious problem  Like an AI only knows what’s out there while many doctors, like some researchers, like research doctors I think, they have ideas about more, more things that are rare, and then I might flag up, I’m not really sure about that.  I, I think there’s certain, I think they can’t really come to that idea unless they, they’ve taken time to research into it and the impact, the potential impact it could have and I think if it’s really worthwhile then they’ll, they’ll see that it is the best interest of patients to pursue it. But if they don’t take interest in it, that’s because they think it won’t benefit patients as much as people are saying, so I think that, that needs to be listened to as well. |
|  | Evidence-based | I think I’d rather wait a bit longer until it’s like a lot of evidence that has been like basically perfected and we can fully trust it. |
|  | Low-risk tasks | Lower risks, sure, just because the waiting time’s not actually crazy so they might help in that case. But nothing like too serious, I personally think. Like I think I need to see a doctor but that’s not a computer. |
|  | Limit AI use | Erm, yeah, just by limiting the use, I guess. Like what you mentioned, like for the doses, like giving, yeah, I think that’s fine, that seems very reasonable, rather than like, here’s what I know, can you please give me, what do you think, like what’s the diagnosis. |
|  | Importance of proper algorithm development | The thing with the CT scan is you need to make sure it’s refined, you know like, it knows what it’s doing. I think I heard like a radiologist coming in, and talking about that at school and he said, yes, the AI did help out finding out which areas had the bleed, which is really good and that does help, but we need to make sure it is refined and it’s at it’s top, it’s working. |
|  | AI to acknowledge its uncertainty | That’s where we might need to recruit other actual people to like see is it something that the AI, maybe the AI could like have a system like this is something we don’t know, and then that could raise up a flag for other doctors could then look into it, like, okay, the AI is unsure about this, what could we do? |
|  | AI performance standards | Yeah, I feel like if it’s going to be, like, the same or worse than a human, you might as well just have the human who’s had like multiple years of like education and training for it.  I think an AI, if that were the case, that you know, it was, you were consulting an AI first, I think it should be able to ask the same questions that a GP would, given the symptoms that you told it. Erm, and then you know, like you can dig more into what’s cause, like what else is going on, because that’s what a GP would do. I think if the AI can’t do exactly what a GP would do, it’s no use. |
|  | AI for treatment more than diagnosis | Yeah, I think so because you know, if you have, erm, like cancer or something or the obvious solution is chemotherapy or, or radiation, like the AI could tell you, obviously it wouldn’t, you know, not in that situation I don’t think they use it, but it, it knows like, it would know the treatment to give and how to manage it and I think that’s more of a knowledge based thing, rather than a thinking based thing. Erm, so I think it could do that, erm, with treatment as long as it was checked. |
|  | AI for monitoring | We already have, I think a friend of mine who, who was diabetic she had like, they have like a small computer thing and it tells them if their insulin’s too lo and it’s like so useful already. |
| Impact on Society | Replacing jobs | there is the worry about like replaces a bunch of jobs. |
|  | Deskilling | Yeah, I think it just decreases the need for human intelligence in, in medicine which is not exactly the way I want to, that I want to world to go. |
|  | AI as divisive issue | I think as well, because AI at the moment is quite controversial and people have so many different opinions on it, it’s really unfair to not tell people, erm, that it is going to be implemented in their care, because some people are completely against it. Like esp-, I think especially the older generations they hate the thing and, and they’d be completely unhappy for it to be in their car so I, at the moment I think they definitely should be telling them and how it’s going to help but otherwise, if it becomes a standard, I think maybe just like a warning, it might be used in your care but… |
|  | Need for human interaction | Erm, I don’t think I would be as comfortable, only because a machine can never really replace a human in, in terms of like emotional impact, er, I feel like, so simple care, not like actual like surgical, for example. Something like that requires more like human interaction, like an AI, I don’t think that, that could ever replace, even if there’s like a thing I’ve heard something about, using AI in machines for therapy, having that connection. I think like I think at the end of the day, I don’t think it will replace a person.  Because they can build more of that trust whereas like, again, the human interaction is so important for that because the patient might not mention certain things to an AI, because they might not think it’s like important but then when you have that person, like the doctor being there with you and then you kind of build this rapport and then to a point like, the, the patient just tells you, oh I also this is a problem, then that might be like a big red flag and that might give you more hints on like what actually is the issue |
|  | Equity | I have questions about accessibility, like who do you think will get AI treatment? It’s just a general question.  […]  Patients but like what kind of patients would you expect to use AI.  […]  And also world wide as well, only certain countries will have the funding for AI technology in their care. |
| Organisations | NHS trusted | Erm, I think I would trust it more if it was the NHS because like there’s such a big institution that if like the AI isn’t like well made it’s like really big on their part. They can get like probably like lawsuits and stuff, so I feel like they kind of have to make it like really, really good. |
|  | Lack of trust in private companies | I think with private companies making it there is more of a risk of them selling data, just because they, they might, you know, there are so many companies that promise to keep your data confidential, they just never do. Erm, so I think, and also they’re more inclined, because they are a private company to sell data for a lot of money, obviously. |
|  | Trust in established non-profits | I would say yes, I would more likely to be trusting them but also it does depend on the, the background they have like if they’re more well known and they have like previous experiences then I would more likely trust them, but if it’s like something new, maybe I will have my doubts till I see like results and evidence and then I would like put my trust in it. |
| AI novelty | Independence from human oversight | Ehhmm, I’m trying to think, no, there is a difference definitely. I think the reason why we’re starting to talk about this a lot more is because of these more popular AI apps that have been produced like ChatGPT, and like, I guess I didn’t make that connection that they do actually use technology every day and we just only recently started talking about it. Maybe it’s because of the site that you mentioned, there was like people overseeing it, maybe people are considering the possibility of it, one day there won’t be a person looking after the AI and the AI will do everything on its own. Maybe that’s why we’re talking more about it. |
|  | Time creates familiarity | I feel I would accept it to be honest, because I feel like so many like technologies that like 20 years ago, people were like this is so new, how are we meant to trust it, but now nobody talks about it because it’s standard now. So like in 20 years that might be the case with AI, and we’re just used to it at that point. That’s kind of normal, we don’t even think about it.  Erm, I don’t know, it’s just, it’s just because it’s so recent that even if it’s proven 99% accurate, people still won’t trust it.  if AI says, er, makes like a really amazing diagnosis and it’s really accurate and it’s 100% correct, erm, I think people are still going to have a bit of doubt in their mind, because you’ve gone from like 0-100 of healthcare being 100% in, you know, in a human’s hand, erm, to not. So, I think there’s ways to ease it into healthcare practices, erm, because I was on a webinar the other day and the doctor said, erm, that they use a specific, erm, website to, er, know how much of a, how much medication they need to give a patient based on their weight, height and things like that, but they don’t know that off the top of their head. They have to use a site for that, of course. Erm, however there could be some AI technology that helps them, like a short cut to get, to help them get it there quicker. Erm, and obviously, erm, you can trial this out by obviously having the, the very trusted site that they use to check that, as a reference point to make sure it matches but that’s kind of quite low risk, like, you know, you could still use AI as, erm, as a way forward in that aspect, erm, but no one’s life is exactly on the line… |
|  | Older people not familiar | I think it just depends on the person, because there’s a lot of people that would probably be really stubborn about it and just completely refuse it. Erm, but I think in general a lot of people are quite open minded about it, er, so, yeah, like I would accept it personally but like my dad, for example probably would refuse it.  They’re just, I think they just don’t trust it like and, I don’t know, I think, especially older people because they’ve grown up all their lives with just, relying on other humans for everything and I think all of a sudden having this AI that’s new, and maybe other people trust it, but it’s just stubbornness I think or… |
|  | At first AI should be voluntary | I think the implementation of it will be really important. I don’t think that it should be implemented and then people should be asked like, okay, this is, you know, the AI is being used for this, are you okay with it. I think it’s just, at least, at, at, at first, like, should be voluntary, erm, er and then maybe, you know, as, as the years go by, like it becomes more of a, you know, if you’re not okay with it, you can go to this other GP but I think the implementation of it is the most important thing, just because there’s going to be a lot of mistrust and a lot of controversy, so I don’t think it’s fair for it to be implemented, just kind of suddenly and, yeah. I think there should be more of an option and a voluntary thing first. |
| AI benefits | AI as less scary than GP | I think a lot of people going to GPs are shamed, so I think in that case, maybe, but it really does depend on your GP I think, like because some of them from what I’ve gathered, you know, are quite comforting to be around and people have no problem with them but also some are scary, erm, so I think it just, yeah… I don’t know if someone just wasn’t comfortable with a person then I think that maybe an AI would be better for, for that interaction but… |
|  | AI can support clinicians | I think with what we’re kind of discussing is the principle of, er, perfecting a certain diagnosis but I think it’s very easy to, erm, for a health care professional to see a scan and make , er, a diagnosis but miss, you know, a certain section of it that kind of changes everything, erm, and it’s a bit, erm, intel, erm, so I think it should be encouraged but like I agree with the point that the doctors are very familiar with certain scans and what they indicate, erm, but I think AI would help enhance focus on certain things. Like a diagnosis that’s harder to make, that more could go wrong. |
|  | Takes away burdens | I would say both, in a way, first of all the minor risk is really good for general, that, that would like take off most of the bur-, like small burdens from the doctors. |
|  | AI as more objective | It would be more objective but it, because obviously it has no bias on gender, I hope, but, erm, it’s just that like you, in your scenario the woman knows everything that’s going on with her, and she’s telling this doctor, and the doctor’s just not listening |
|  | AI for speeding things up | I personally will still trust the human, but again it’s who, who’s available most. If the AI’s like right there and I can access it easily, maybe that’s a factor, some people will consider the accessibility with an AI, compared to a doctor  Yeah, because obviously the NHS at the moment, everyone’s waiting for years or something just to get anything done. Erm, so ideally an AI would speed up things like that but it’s just, I, I, at the moment I think no one would, yeah, again no one would, yeah, people would prefer a human, so you know, so maybe somewhere further down the line it could speed things up  It’s better than waiting for like three days, yes. |
|  | Can help with common things | There’s also like some serious things that are, that, you know, are, are very common like cancer for example, I think an AI could probably do quite well with cancer, just because it’s so common, erm, and we know quite a lot about it I think so in that situation I think it would be fine, despite the severity of cancer, obviously |
|  | AI can help in training healthcare professionals | I was like in this other webinar, er, regarding AI and health. I think it really does help, like nowadays, like medical students, I saw like a scenario where there was like an AI bot who acted as patients and they gave like the patient the doctor, the medical student histories and everything and then based on that the medical student then uses their own critical thinking to then diagnose, so it’s like fake patients almost that could help in training processes. |
|  | AI help with staff shortages | I think it would be useful just because there’s so many like staff shortages at the moment and the waiting times for the NHS are just ridiculous. But, erm, I think the ability to see how serious a patient problem is before booking an appointment would be useful, erm, because there are so many people going for appointments when they don’t need them. |
| In care of others | Same broad attitude | I think it would be more or less the same |
|  | Awareness of other’s opinions | I wouldn’t want to say yes to it if I knew that they wouldn’t want it, even if I wouldn’t mind myself. |
|  | Best interest | Whatever’s best for the child, then.  Absolutely not. no. He wouldn’t want it, it’s not fair.  […]  It depends on how much it could help, because if it was just speeding it up or something I, I’d still say no, but if it was like the AI can definitely do it, and there’s absolutely no chance that people can, then I would say yes. I wouldn’t be happy about it but, I, I, in that situation you’re kind of forced to. |

**Retirees**

| Theme | Sub-theme | Example |
| --- | --- | --- |
| Governance | Inform about the use of AI | Yes. [response the question ‘do you think that patients should be told when such technologies are used in, in their care?’] |
|  | Transparency about AI development | That would be, er, helpful, yeah [response to ‘Yeah, so, so we, we had some responses that, erm, that suggested that however, that the process should be very transparent so you’re able to have somebody else to come and, and check basically on the process, see where they got the data, data from, what did they do with the data and if the story matches and obviously whether it works at, at the end of it. So, so if you have your worry with regards to that, is it all about testing and making sure that, that the people who developed it are accountable and you, would that reassure you, if there were like some specific processes in place to test whether, you know, somebody…’] |
|  | Assessing the AI | I’d be inclined to try and make the best of it but at the same time I think you do have to be a bit careful about getting a bit too blasé and maybe not keeping an eye of how well it’s performed and we need to make sure that, erm, it’s performing to, to it’s best level. Erm, if you get a glitch, you know, within something that’s supposed to discern the best possible treatment for a given condition or a given patient, if you get a glitch in that, erm, it could be very nasty.  Like who monitors it all  Yeah, and presumably there would be some ongoing audit of, of performance? |
|  | Assigning culpability | Yeah, but if you, er, make AI culpable, nobody is culpable. |
|  | Ethical oversight of AI research | Would there be any sort of ethical thing, mixed up in this? |
|  | Safeguards | I suppose like most of us I would like to be sure that there were some safeguards at least to try to make sure that nothing absolutely dreadful went wrong. There’s always going to be a hiccup occasionally, nothing in life is perfect, but, you know, you wouldn’t want a really big malfunction to cause trouble or, you know, hacking or, or anything else |
| Data | Security | Theft of data.  […]  There’s so much hacking these days. |
|  | Privacy | There’s a court case going on now, in America. Erm, I can’t remember the guy’s name but he’s some multi-million pound company owner and he had promised, erm, his customers or clients that their biological data would not be sold or shared and that they could at, at any time, erm, delete their data, they, they’re given a site they can go to and they could delete it, whenever they wanted to and, and now he’s in trouble because this, this character, whoever he is, I can’t think of his name, erm, he is actually trying to sell the biological data of millions of his American customers. Even though, under his, erm, consent form, and, you know, all the blurb that people are supposed to read, in very, very tiny writing, he put down that even if people deleted their data, erm, and, erm, disabled or cancelled their, er, place on the site that the, the data would still belong to the company and it could still be used any way they chose. So, he lied to all these people and it was only if they had the patience and the good sense to read the very, very tiny print that they discovered they were being taken for a ride, so now this man is up to his neck in trouble. Good, I hope they sue the pants off him. |
|  | Collected beyond standard needs | Well that’s what I was wondering, you know, how much detail, how much actual data is it more than we would normally have for a patient’s ordinary medical records. |
|  | Training data falsification | Erm, if they make up, if they have a particular drug that they want to push forward and they, they add data to make sure that that particular drug is going to, erm…  If somebody’s cooking it. |
| Organisations | NHS trusted | I would trust the NHS, I’m not quite sure I trust the government and I certainly wouldn’t trust these great big corporations like this fellow who’s getting skinned alive in America. |
|  | Government not trusted | I would trust the NHS, I’m not quite sure I trust the government and I certainly wouldn’t trust these great big corporations like this fellow who’s getting skinned alive in America. |
|  | Corporations not trusted | I would trust the NHS, I’m not quite sure I trust the government and I certainly wouldn’t trust these great big corporations like this fellow who’s getting skinned alive in America. |
|  | Academia is liked | Or academia |
|  | Not-for-profit more acceptable | Yes, I think that would be more, I think you’d feel less, less concerned about the possibility of a bias because if there’s no profit involved, there can’t be a profit motive. |
| AI novelty | Worry of novelty | I don’t know, I know very little about it, but, erm, I, I think it’s just all such a new, maybe it’s not, it’s only new for me, I don’t know, but I don’t know, potentially where, I don’t know, where things could go wrong  I just don’t know anything about it, really but, whenever I have heard about it, it’s actually been quite negative. |
|  | Older people might adjust slower | Dinosaur thoughts probably but… |
|  | AI not gaining control | is the whole point of making AI, AI a tool rather than something that controls. |
|  | Becoming familiar with AI changes attitudes | PA3: Can I ask you a question. Erm, whilst we’ve been talking, I’ve been thinking is this a bit like, so when you have a Cochrane Review, you know, you go through all the papers that are available…  PA3: And, you, you come up with the best practice something. This is kind of the computer equivalent of a Cochrane review isn’t it?  PA4: I suppose that’s how I’m seeing it.  I must admit you’ve, I came in feeling very sceptical about all of this and now I feel, oh, there’s a lot more to it and it makes a lot more sense because it’s just got more information  I mean I’m generally an optimist regarding AI, but I’ve been made more aware of some of the things I should be a bit cautious about. |
| Human element | Trust in people | Well I think we want to believe that the NHS is, I mean yes, of course there are cases where things don’t go, you know, perfectly, but I think generally the medics I’ve known, I’ve trusted  I think I generally trust a person more than I’d trust this, whatever it is, this big something, so, I, I don’t know  I would be happier with a medic who said, right, you know, I’ve looked at your case, I’ve run this through AI which suggests this would be the better outcome, I kind of agree with that, how do you feel about it. That would be the approach I would be happier with rather than simply, well AI says this, therefore that will, because I like that human element of judgement which I think is, certainly with what I’m doing |
|  | Human input can cause errors | But the, the AI is, the input is human, the input into AI is from human beings as well so, so you still have the element of error. |
|  | People can see more context than AI | Because all of these examples are going into a pot and a you get a standardised version from all the information that you have, but you’re bound to get somebody, somebody that doesn’t fit, quite fit into that.  And the mums who would say, oh I’m not sure, you get, I always have to go, have a look, this kid would be moribund and then another kid with another mother saying, oh, he’s terrible, he’s terrible, you couldn’t get hold of him to examine him.  […]  It’s a holistic thing isn’t it? |
|  | Patient-clinician relationship | PA1: Well if they weren’t happy fundamentally then …  PA4: Yeah, well then you haven’t got a good doctor/patient relationship to work with, have you.  Yeah, eye contact, not just a chatbox, but actually that interpersonal communication. I, I think it’s very important in healthcare  And when, when you feel ill, you feel very vulnerable so the sense that somebody is acknowledged you and your concern is actually therapeutic in itself? |
|  | Telemedicine | And I absolutely hate chatboxes.  It’s got to be face to face, there’s no substitute for a real human being.  PA4: Yeah, it has it’s advantages [responding to a comment about telephone consultations]. When I had, when I had shingles I didn’t need to see anybody. I’ve got shingles, I just wanted some…  PA1: It needs to be a hybrid, doesn’t it, you need to be able to have both.  PA4: Yeah, so it’s good for something |
| Using AI | Advantage in area of massive knowledge progress | Yeah, and as you say, as a medic it’s hard to keep up with all the late-latest of what’s happening and, and it’s, as he says, getting more and more complex so it would, it would be an advantage to be able to use it in health care. |
|  | Personalised care | Well I suppose I’m quite optimistic as I can see the, the idea of taking patient characteristics and you’re more likely to benefit from one treatment than another. I think that would be quite a positive thing.  […]  I’m just thinking, erm, the, the work they’re doing on genetics and, erm, drug tolerance, especially with opioids and things like this… |
|  | Protect from human mistakes | But, erm, we know from, from reports that scans have been misread by human beings, when you were tired or… |
|  | Should be freely challenged by humans | But again, you need ability for a human to make their own judgement and override it because patients vary, so okay, you present at this stage but disease progression can vary, so if my patient is getting deteriorating quite quickly, although maybe when they first presented it wasn’t so urgent. I would need to be able to say hang on, this patient isn’t behaving as the average, they, they need to be seen sooner.  if the patient doesn’t agree with that?  […]  If, if the doctor doesn’t agree with it?  […]  … and maybe if they’ve got a valid enough point, you know, I don’t know what’s happening there…  I suppose I’ve always thought, as a doctor, I’m responsible for the advice I give but I’m not responsible for whether or not that patient accepts or follows it. So the responsibility remains with the patient. AI suggests this, the doctor suggests that, the patient doesn’t want either of them and it’s my job as a doctor to say, to explain difficulties in following what the patient wants to do but ultimately they retain the, the responsibility… |
|  | Predicting prognosis | I mean that would be interesting if AI could predict prognosis, because that was one thing I found very difficult when I was working and relatives would ask me how long  […]  And I’d say, well I can tell you the average but, erm, so if, if there’s a way for predicting for an individual what their specific prognosis was, I’d be extremely impressed with AI. |
|  | AI mistakes affect more people | Can I ask, does a small mistake like magnify, or multiply, or does that make any sense to you. Have I, I have got the idea all wrong, if you feed in something does that then, and if it was wrong would that have a huge effect, rather than, than an individual clinician |
|  | Saves time | To do it better, and more, a lot more quickly. Yeah, and getting hold of paper notes and data and working through it is a slow process but AI can just in a few second access… |
|  | Same principles for proxy decisions | If I accepted it for myself then I can accept it for everybody else as well, because like I love myself the most. |
|  | Respecting people’s wishes | Even though I didn’t agree with it I would respect their wishes.  […]  I think that would be difficult, a difficult problem. Erm, but overriding their wishes would, I’m thinking if somebody hadn’t got capacity…  PA4: Overriding their wishes would be difficult.  PA1: But people are going to be anti AI, what, what do they really know…  PA4: Yeah, and what do they understand… |
| Treatment recommendation | Economic factors | though even if they rubber stamp this thing, the AI, surely there’s an awful lot of economics involved in all this, as well. Even if they say this is what is best for you, rather than that procedure. If, if they can’t afford to do it, there might be cheaper  I think we have to accept that, that health care does have to rationed. I mean we can’t have what we want, when we want it, irrespective of cost.  PA4: But yeah, but in a way I’d like to think if I was a patient you would choose the most effective treatment for me, rather than…  PA1: Yeah.  PA3: Cost effective.  PA4: … the cost effective, er, but that would be as an individual rather than thinking about the population…  PA1: I think that would speak for everybody.  PA4: Well we have to think about the population as well, because we’re members of a population. |
|  | Conservative recommendations | I suppose it’s like, like a doctor, do you prefer a doctor who’s gung-ho or somebody who’s a bit more concerned, yeah? I suppose I tend to go on the conservative side. |
|  | Some recommendations fundamentally unacceptable | Because if you’ve got an elderly relative who’s got cancer and they self-diagnose and say well you’ve got six months to live, so we, we think that you should have a pod and go to the cemetery |
